# Supplementary material for: Mouse-Derived Isograft (MDI) In Vivo Tumor Models II. Carcinogen-Induced cMDI Models: Characterization and Cancer Therapeutic Approaches
Source: Cancers (Basel). 2019 Feb 19;11(2):242. doi: 10.3390/cancers11020242 (PMC6406786; doi:10.3390/cancers11020242)
Supplement: Supplementary file 1 [file cancers-11-00242-s001.pdf]

cancers

MDPI

Type of the Paper (Article - Supplementary Data)

Mouse-Derived Isograft (MDI) *in vivo* Tumor Models

II. Carcinogen-Induced cMDI Models: Characterization and Cancer Therapeutic Approaches

- Supplementary Data -

Janette Beshay [1,3a], Peter Jantscheff [1a*], Thomas Lemarchand [2,], Cynthia Obodozie [1], Christoph Schächtele [1], Holger Weber [1*]

[1]*In vivo* Pharmacology, ProQinase GmbH, Breisacher Str. 117, 79106 Freiburg, Germany, peter.jantscheff@t-online.de; Janette_Beshay@web.de; c.obodozie@proqinase.com; c.schaechtele@proqinase.com; h.weber@proqinase.com

[2]TPL Pathology Labs, Sasbacher Str. 10, 79111 Freiburg, Germany, lemarchand@tpl-path-labs.com

*Correspondence: peter.jantscheff@t-online.de; phone: +49-7666-913-0396; h.weber@proqinase.com; phone: +49-761-769996-1733

[a]Peter Jantscheff and Janette Beshay contributed equally to this work. [3]present address JB: Charles River Discovery Research Services Germany GmbH, Am Flughafen 12-14, 79108 Freiburg, Germany

Received: date; Accepted: date; Published: date.

1. Supplementary Data

Suppl_data_cMDI_models – Cancers.docx – containing:

1.1 Doc. S1-cMDI: sMDI - Histological and Pathological Analysis p 02

The document summarizes detailed histopathological analysis of primary and derived tumors of various cMDI models inclusive respective large microphotographs

1.1 Doc. S1-cMDI: cMDI - Histological and Pathological Analysis

The document summarizes detailed histopathological analysis of primary and derived tumors of various cMDI models inclusive respective large microphotographs

Table of Contents

1.1 Doc. S1-cMDI: cMDI - Histological and Pathological Analysis 2
List of Figures.. 2
JA-2011 cMDI: NOS sarcoma composed of spindle shaped elongated cells... 4
JA-2019 cMDI: NOS sarcoma - storiform pattern... 8
JA-2041 cMDI: NOS sarcoma composed of interlacing bundles 11
JA-2042 cMDI: NOS sarcoma with hemangiosarcoma-like differentiation... 14
JA-2034 cMDI: NOS sarcoma within adipose tissue of the panniculus 17
JA-2043 cMDI: NOS sarcoma with varying aspects from well differentiated to anaplastic................. 20
JA-2044 cMDI: Spinocellular carcinoma (epidermoid carcinoma) 25
JA-2017 cMDI: NOS sarcoma varying from well differentiated to moderately anaplastic; no giant cells 28

Note: c-MDI is "carcinogen-induced Mouse Derived Isografts; NOS is "not otherwise specified"

List of Figures

Figure 1: Localization of tumor/suspicious tissue in tumor JA-2011 4
Figure 2: An example of one of the cutaneous masses, ulcerating the skin and deeply invading the adjacent tissues from index case 2011-16 (#11975 tu)... 4
Figure 3**:** phenotypically stable sarcoma tumor model: transmitted tumor to mouse 0242-17 5
Figure 4: Established c-MDI tumor **0242-17** from index case **2011-16**... 6
Figure 5: Comparison of the source **2011-16** (**A)** and derived **242-17 (B)** neoplasms....................... 7
Figure 6: Different macroscopic aspects of the tumors JA-2019 8
Figure 7: Low magnification (sub-gross aspects) of the tumor masses: **A** and **C** index tumor **2019-16** and **B**: established daughter tumor **174-17**... 8
Figure 8: Comparison of the source **2019-16 (A)** and the derived **174-17** (B) neoplasms.............. 10
Figure 9: Different macroscopic aspects of the tumors JA-2041 11
Figure 10: Chemically-induced neoplasms (study #11975): sarcoma, not otherwise specified, male CBA/J mouse 2041/16, MCA s.c.. 11
Figure 11: Chemically-induced neoplasms (study #11975): sarcoma, not otherwise specified, male CBA/J mouse 1607/16, MCA s.c.. 13
Figure 12: Low magnification (sub-gross aspect) of the tumor masses: **A** index tumor 2042-16 and **B**: established daughter tumor 124-17... 14

Figure 13: Comparison of different regions of index **2042-16** and derived **124-17** 16

Figure 14: Chemically-induced neoplasms (study #11975): sarcoma, not otherwise specified, male CBA/J mouse 2034/16, MNU s.c. 17

Figure 15: Chemically-induced neoplasms (study #11975): sarcoma, not otherwise specified, male CBA/J mouse 0125/17, MNU s.c. 18

Figure 16: Macroscopic aspects of the tumor JA-2043 20

Figure 17: High magnification aspects of the index tumor 2043-16: anaplastic 20

Figure 18: Low magnification (sub-gross aspect) of the tumor masses of the index tumor **2043-16**... 22

Figure 19: High magnification aspects of the index tumor 2043-16: fusiform aspects.................... 23

Figure 20: Sub-gross and higher views of grafted JA-2043 into 0074-17 mouse. Tissue similarity with top index tumor at these magnifications is striking. N is central ischemic necrosis of the tumor 24

Figure 21: Chemically-induced neoplasms (study #11975): spinocellular carcinoma, male CBA/J mouse 2044/16, MCA s.c. 25

Figure 22: Chemically-induced neoplasms (study #11975): spinocellular carcinoma, male CBA/J mouse 1418/16, MCA s.c. 26

Figure 23: Originator tumor of the JA-2017: **2017-16** (see next page).. 28

Figure 24: Low magnification aspect of the derived **tumor 1573-16** grafted with **JA-2017** 0

Figure 25: Tumor **1573-16**: High magnification view of the region in the Green square.................... 1

Figure 26 : Tumor 1573-16: Medium magnification view of the region in the large blue rectangle from figure 24.. 2

JA-2011 cMDI: NOS sarcoma composed of spindle shaped elongated cells

JA-2011/0242-17: NOS sarcoma composed of spindle shaped elongated cells

Figure 1: Localization of tumor/suspicious tissue in tumor JA-2011

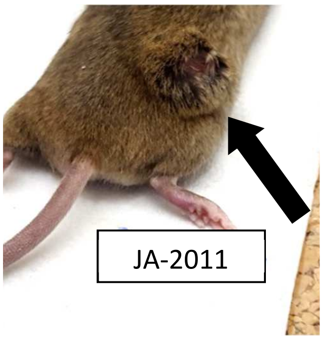

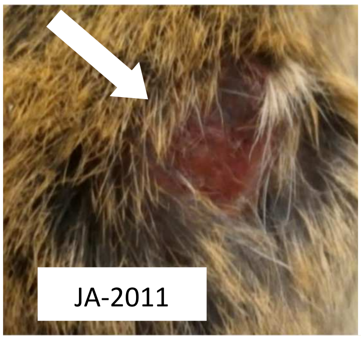

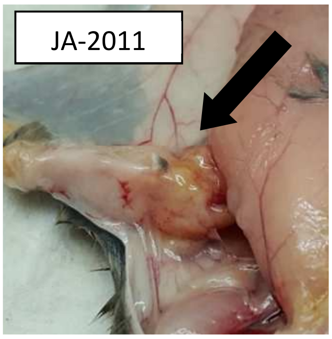

Figure 2: An example of one of the cutaneous masses, ulcerating the skin and deeply invading the adjacent tissues from index case 2011-16 (#11975 tu)

The sarcomatous tissue fully invades the dermis and widely dissects out the preexisting dermis (left inset), eliciting focal epidermal hyperplasia and ulceration at tumor center (black arrows). This is one of the 3 fragments examined

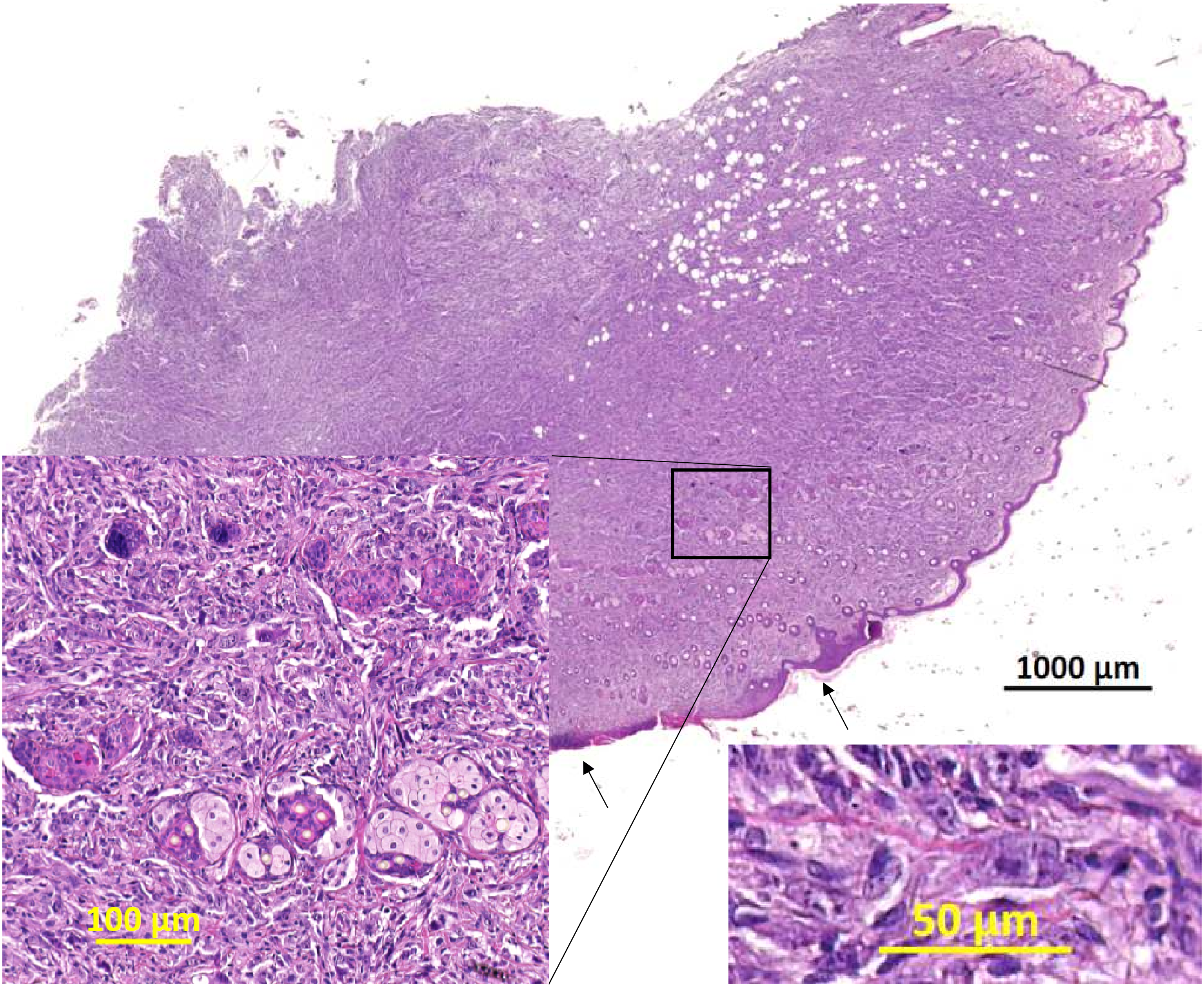

Figure 3: phenotypically stable sarcoma tumor model: transmitted tumor to mouse 0242-17

There is an apparent increase of anaplasia, with the emergence of more numerous (higher density) of large, anaplastic mono-nucleated (red arrow in lower inset) or poly-nucleated giant cells (see the **black arrows** in insets). See also Figure 4 and Figure 5 narratives on next pages.

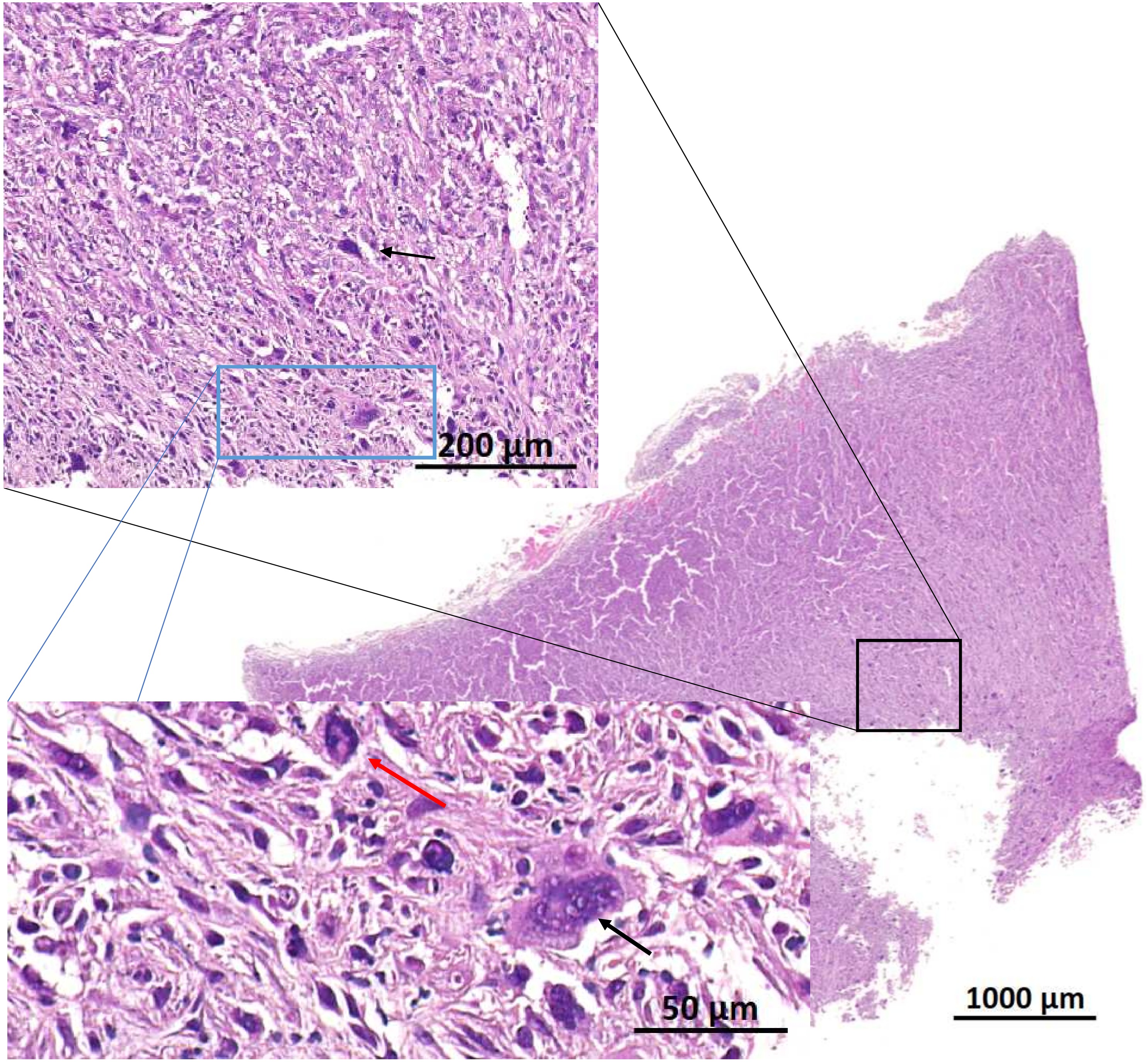

Figure 4: Established c-MDI tumor **0242-17** from index case **2011-16**

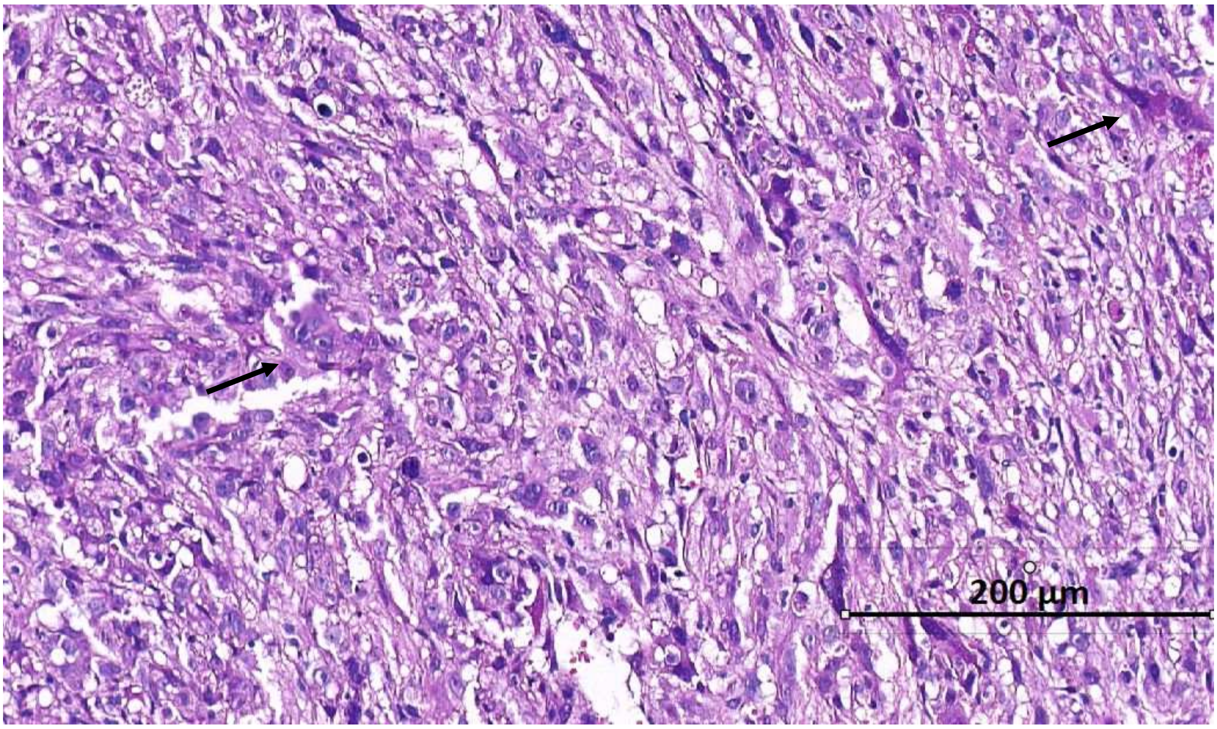

Mouse ID #242-17; from index case: mouse ID number 2011-16. Strain CBA/J ♀.

Sarcoma, not otherwise specified (NOS) composed of spindle-shaped, elongated cells (neoplastic mesenchymal cells), with numerous giant mononulear or multinucleate giant cells or "anaplastic cells" (**black arrows**), suggesting the evolution towards an anaplastic sarcoma. IHC markers could be useful to further characterize this tumor.

Morphological diagnosis: Sarcoma NOS.

Comment: the most common MNU-induced malignant neoplasia

Image source ID: 2017-10-24 17_25_38-12551_242-17___Establish.czi - ZEN 2.3 lite

Figure 5: Comparison of the source **2011-16** (**A)** and derived **242-17 (B)** neoplasms

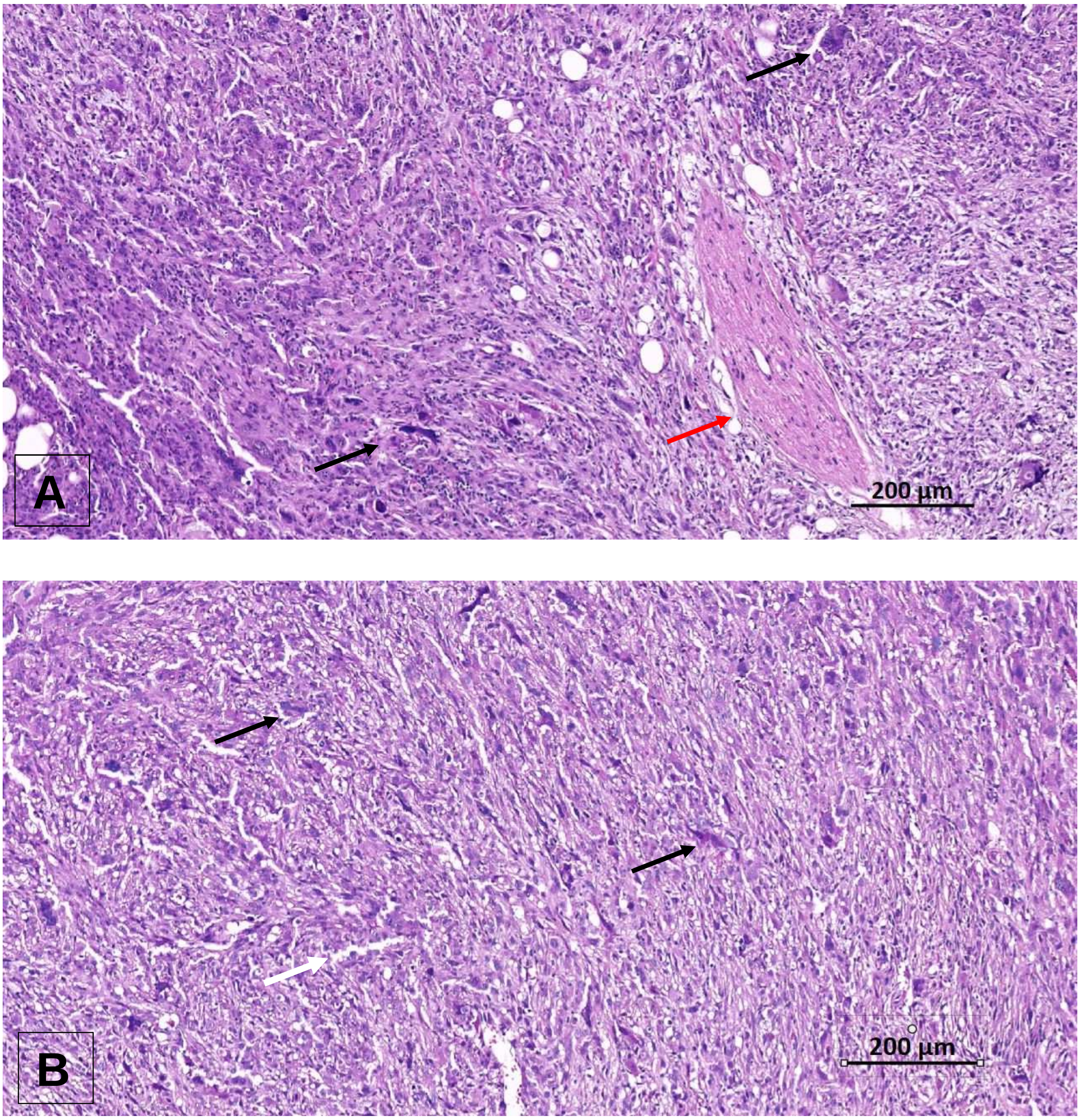

From index case: mouse ID number 2011-16. Strain CBA/J ♀. Induced with MNU.

Sarcoma, not otherwise specified (NOS) composed of spindle shaped elongated cells (neoplastic mesenchymal cells), with numerous giant mononulear or multinucleate giant cells (**black arrows**), and deeply invasive, in this field invading a host nerve bundle (**A**: red arrow). Notice the remarkable phenotypic stability, at least morphologically with H&E stain, when comparing side by side at matching magnification the index **(A)** and derived **(B)** neoplasms. White arrow: artefactual tissue crack:

A: Image ID: 2017-10-24 17_29_36-11975_tu___2011-16___2011-16.czi - ZEN 2.3 lite – index case (mouse ID) **2011-16; B**: image ID: 2017-10-24 17_30_44-12551_242-17___Establish.czi - ZEN 2.3 lite – ID #**242-17**: Extracted from TPL Study Phase Number 780/17

JA-2019 cMDI: NOS sarcoma - storiform pattern

JA-2019/0174-17: NOS sarcoma - storiform pattern composed of interwoven basket weave-like or interlacing bundles of neoplastic spindle-shaped cells

Figure 6: Different macroscopic aspects of the tumors JA-2019

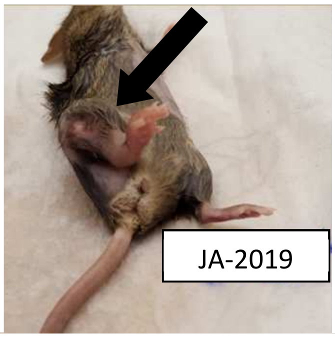

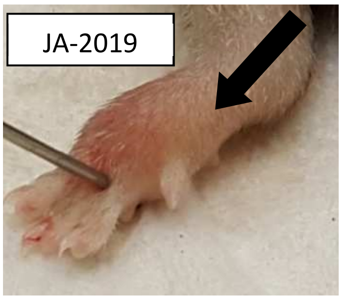

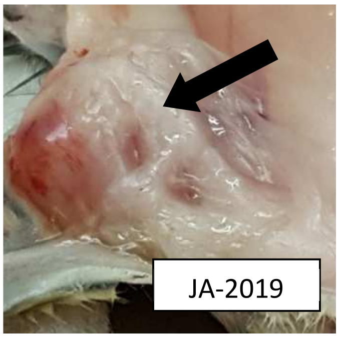

Figure 7: Low magnification (sub-gross aspects) of the tumor masses: **A** and **C** index tumor **2019-16** and **B**: established daughter tumor **174-17**

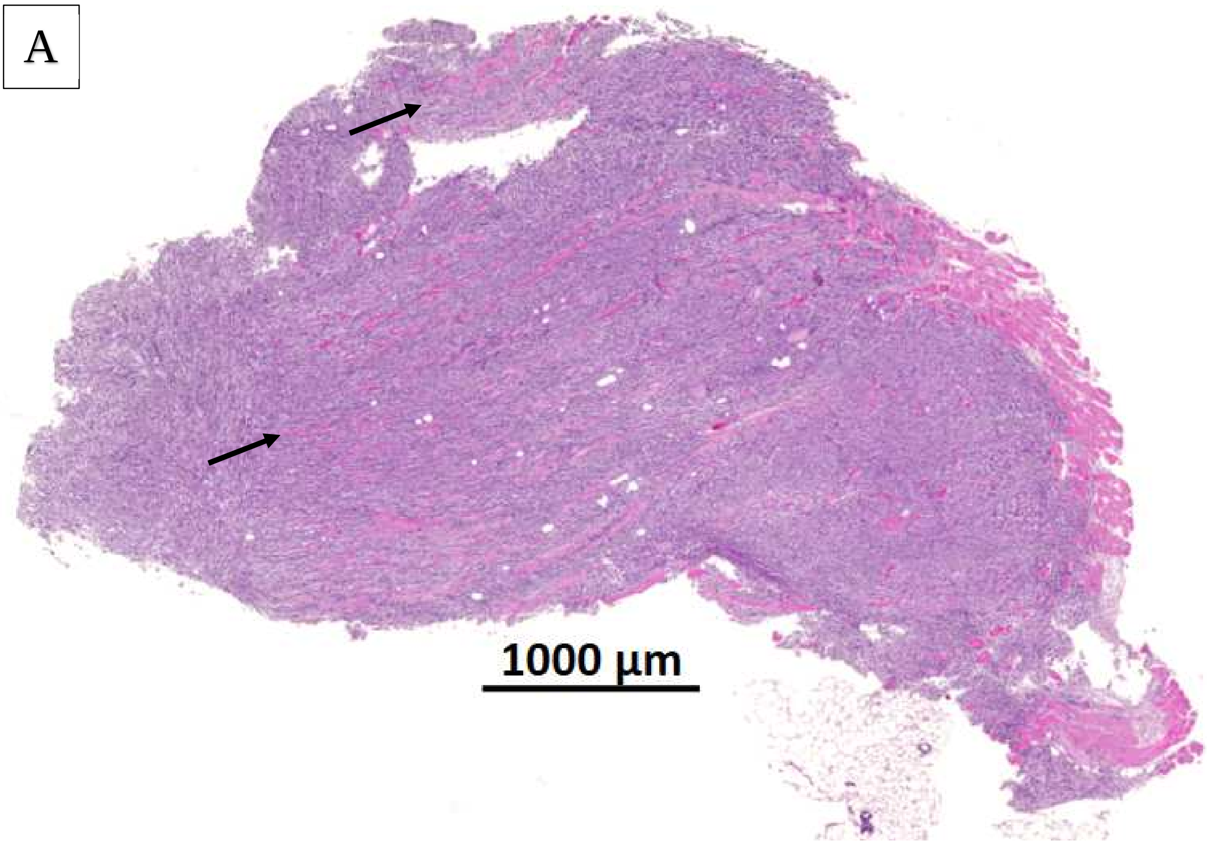

A: 2019-16: The muscle fibers (**arrows**) are widely separated by bundles of invading sarcomatous tissue, without evidence of any characteristic differentiation.

This is 1 of the 2 fragments submitted. The other fragment is depicted in **C**. Both fragments have a similar morphology

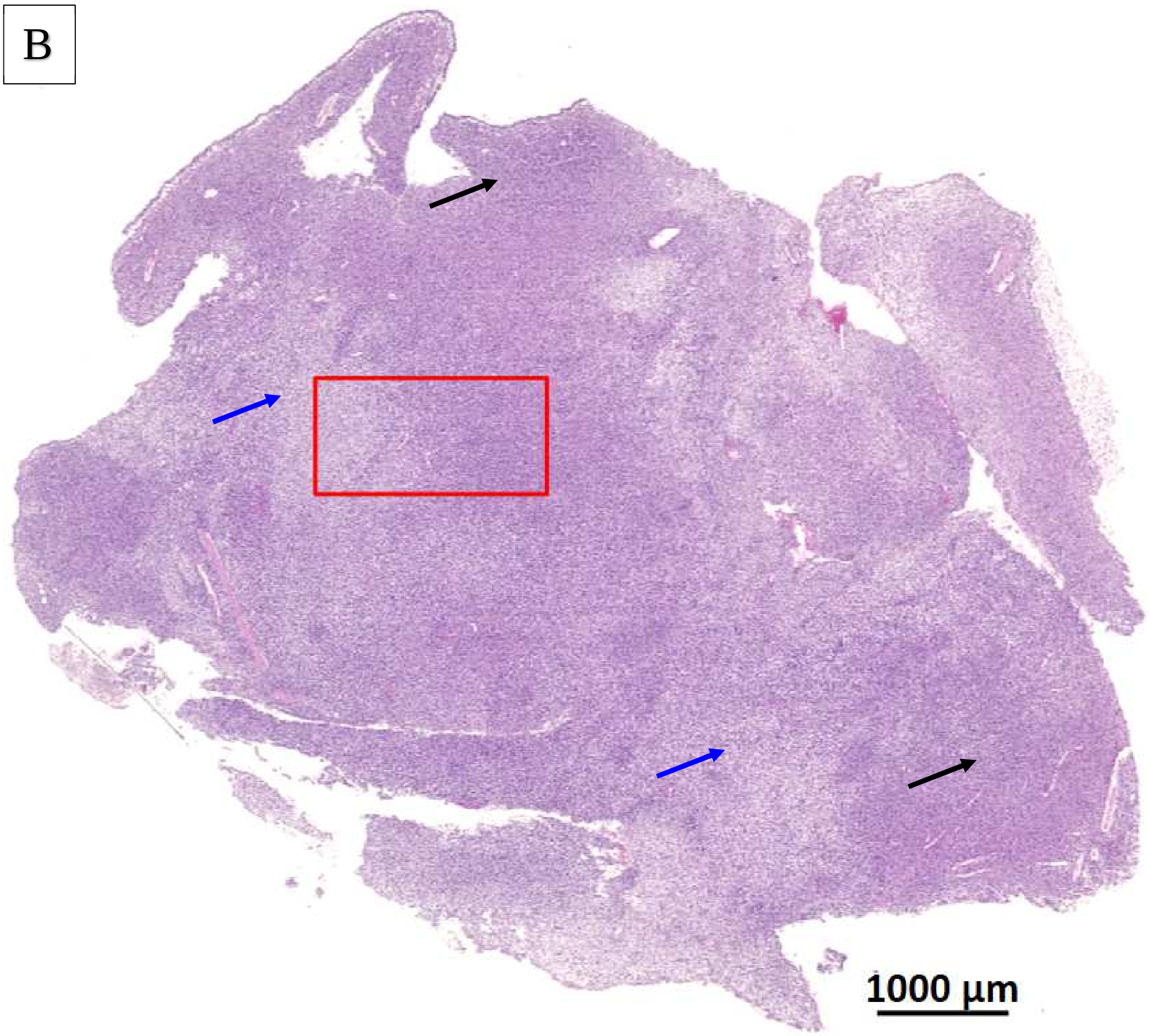

B: Sub-gross view of grafted JA-2019 into **174-17**, showing 2 distinct cell density aspects, a higher, more basophilic aspect (**black arrow**) and a lower paler and pinker aspect (blue arrows) corresponding to lower cell density with also possibly paler cytoplasm.

The red rectangle corresponds to the field depicted in Figure 8 B

File source: C:\Users\TLEMARCHAND\Documents\ProQinase-Paper-Project\neue bilds\2019-01-24 14_59_17-#12551 174-17.czi - ZEN 2.3 lite.png

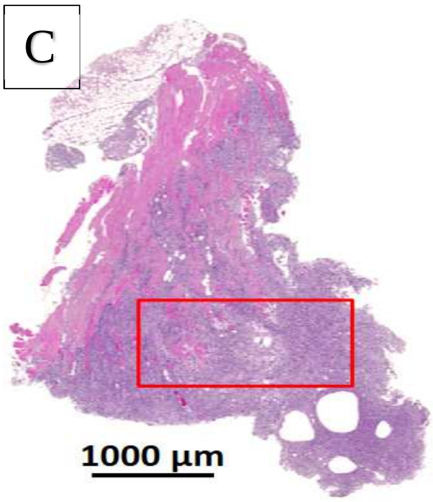

C: Sub-gross view of the sarcomatous tissue fully invading the adjacent skeletal muscles. This is one of the 2 fragments (Fragment 2) examined of the index tumor. Fragment 1 is examined in **A**.

The red rectangle represents the field depicted in Figure 8 **A**

Figure 8: Comparison of the source **2019-16 (A)** and the derived **174-17** (B) neoplasms

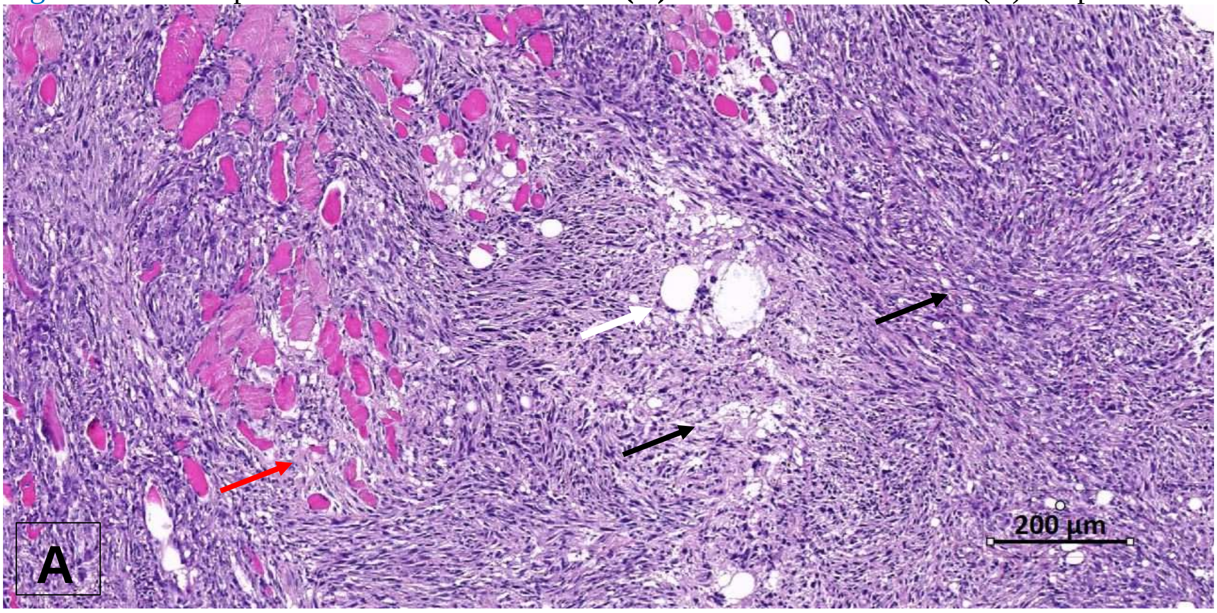

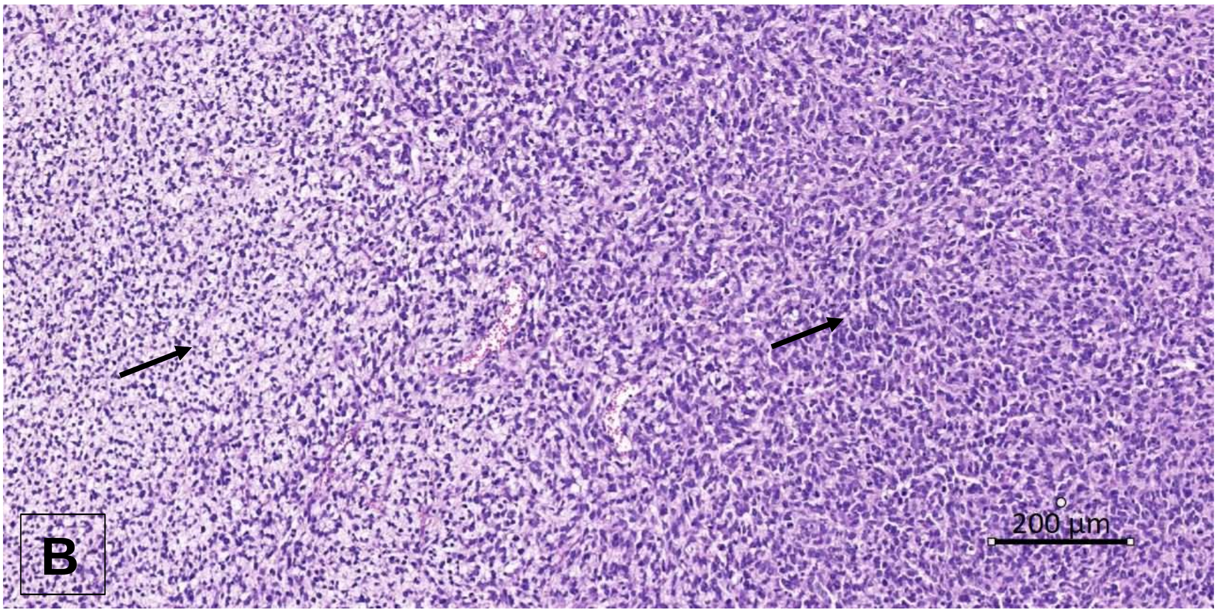

From index case: mouse ID number 2019-16. Strain CBA/J ♀. Induced with MCA. Sarcoma, not otherwise specified (NOS) composed of interwoven bundles of spindle-shaped elongated cells (neoplastic mesenchymal cells), with minimum anaplasia and clearer and denser areas (**black arrows**). They are deeply invasive, in both skeletal muscle (**A**: red arrow, inducing fiber atrophy) and adipose tissue (**A**: white arrow). Notice the relative phenotypic stability, at least morphologically with H&E stain, when comparing side by side the index **(A)** and derived **(B)** neoplasms.

A: Image ID: 2017-10-24 17_32_11-11975_tubein___2019-16___2019-16.czi - ZEN 2.3 lite – index case (mouse ID) 2019-16; **B**: image ID: 2017-10-24 17_32_57-12551_174-17___ Establish.czi - ZEN 2.3 lite.jpg – derived tumor ID #174-17: Extracted from TPL Study Phase Number 780/17

JA-2041 cMDI: NOS sarcoma composed of interlacing bundles

JA-2041/1607-16: NOS sarcoma composed of interlacing bundles

Figure 9: Different macroscopic aspects of the tumors JA-2041

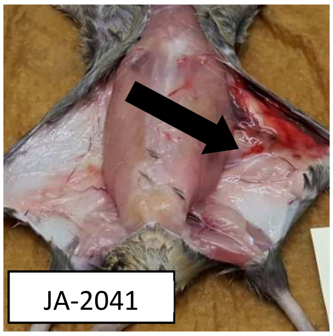

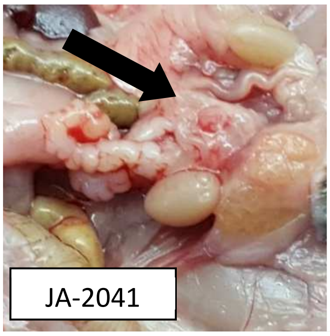

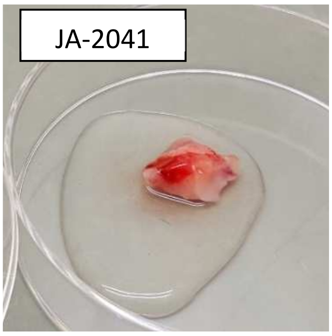

Figure 10: Chemically-induced neoplasms (study #11975): sarcoma, not otherwise specified, male CBA/J mouse 2041/16, MCA s.c.

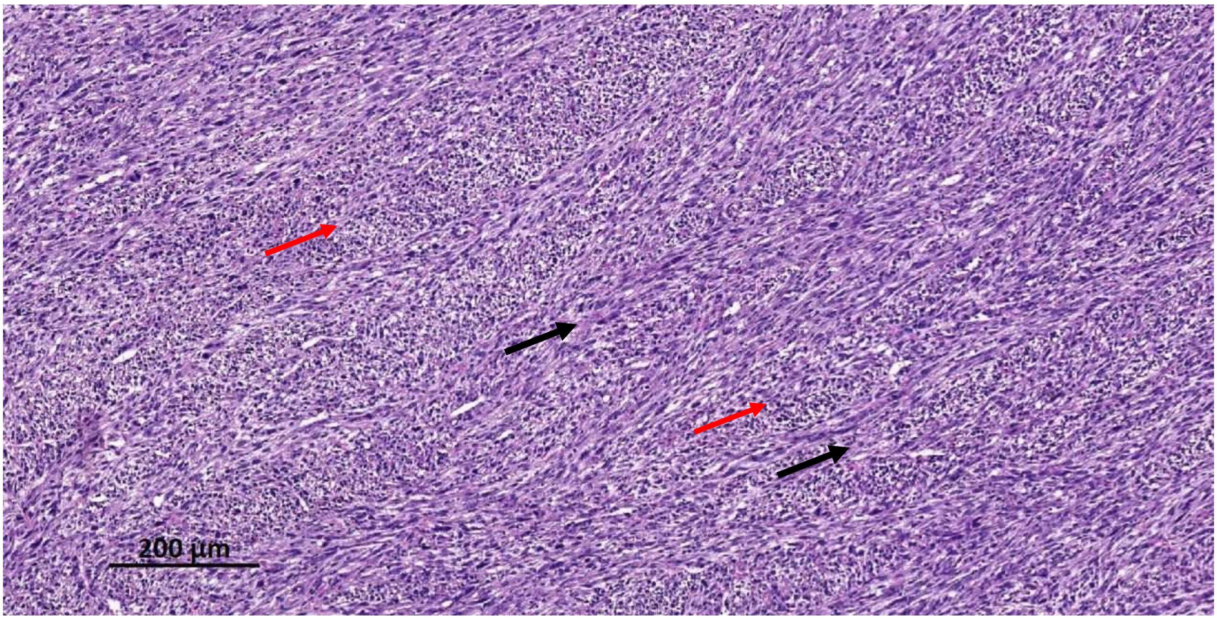

Source: 2017-10-25 13_48_48-TPL811_17_HE_11975_TU_2041_16_4_10_16.czi - ZEN 2.3 lite

Index case: mouse ID number **2041-14**. Strain CBA/J sex: male (♂).

Sarcoma composed of interlacing bundles alternating longitudinal (**black arrows**) and transverse bundles (**black arrows**) or long, rather well differentiated spindle-shaped cells. There is no ischemic necrosis on the section examined.

Morphological diagnosis: Sarcoma NOS, MCA-induced, CBA/J mouse.

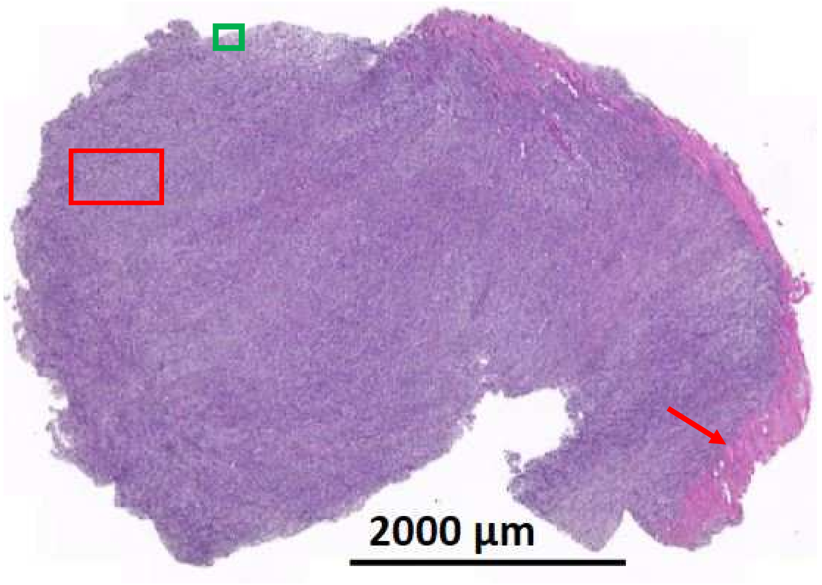

The red rectangle indicates the part of the tumor, which is depicted above.

Notice the homogenous sarcomatous tissue, infiltrating the pre-existing skeletal muscles (red arrow) on the edge of the sample.

Source photo file: 2018-01-23 18_36_54-TPL811_17_HE_11975_TU_2041_16_4_10_16.czi - ZEN 2.3 lite

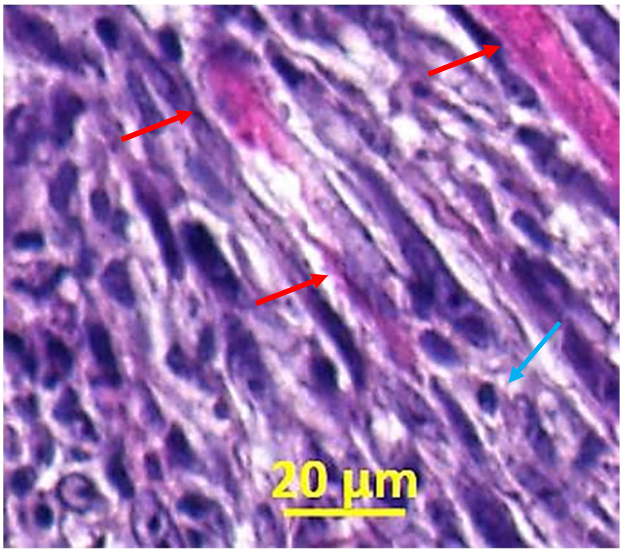

This detail of the neoplastic proliferation is located in the green rectangle of the topographical photo above

The neoplastic cells exhibit low pleomorphism but have numerous infiltrating lymphocytes (**blue arrow**) and deeply invade between atrophic muscle fibers (red arrows).

Source photo file: 2018-01-23 18_45_19-TPL811_17_HE_11975_TU_2041_16_4_10_16.czi - ZEN 2.3 lite.jpg

Figure 11: Chemically-induced neoplasms (study #11975): sarcoma, not otherwise specified, male CBA/J mouse 1607/16, MCA s.c.

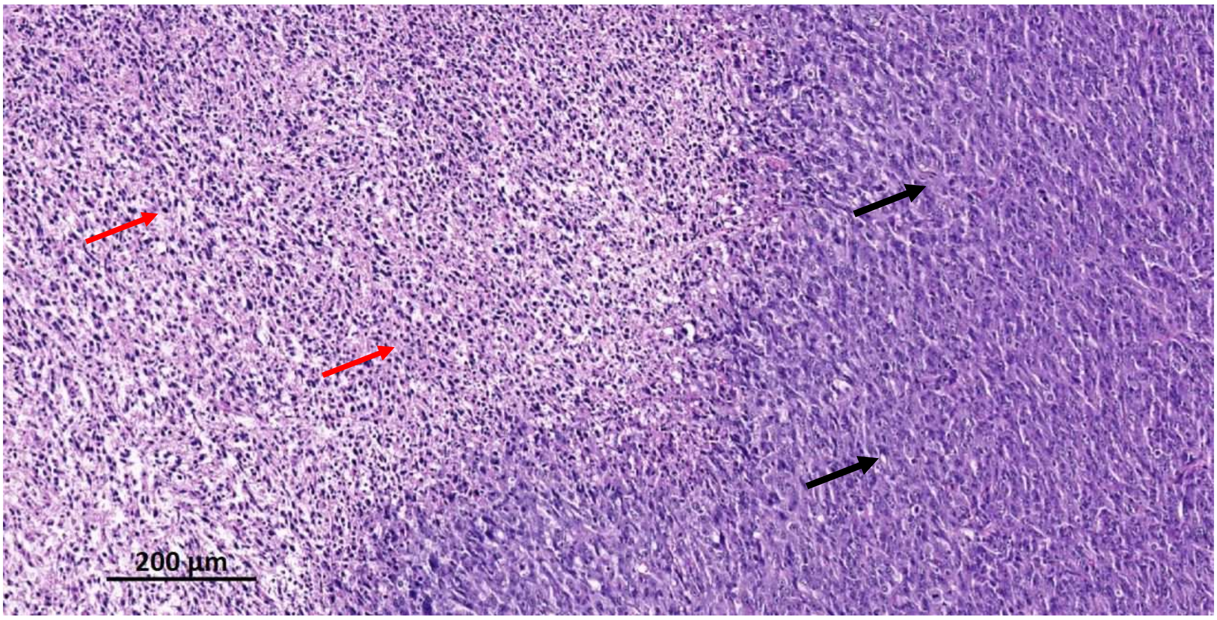

Source: 2017-10-25 13_46_58-TPL811_17_HE_11975_TU_1607_16.czi - ZEN 2.3 lite

Index case: mouse ID number 2041-14. Strain CBA/J sex: male (♂).

The invaded tissue type is unrecognizable (it invades muscle and fat). The sarcoma is composed of poorly intertwined bundles of blastic deeply basophilic roughly spindle-shaped cells (**black arrows**). When compared to the originator neoplasm (see Figure 10), the main differentiating feature is presence of wide ischemic necrosis indicative of inadequate vascularization (red arrows). Lymphocyte infiltration is very dense.

Morphological diagnosis: Sarcoma NOS, MCA-induced, CBA/J mouse.

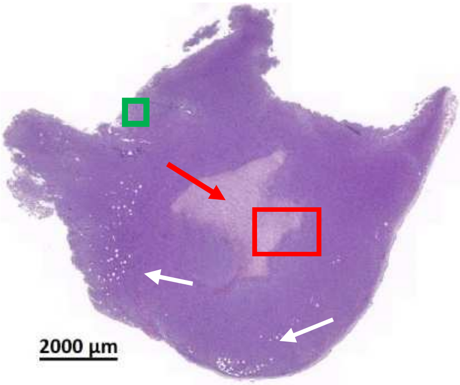

Source photo file: 2018-01-23 18_15_27-TPL811_17_HE_11975_TU_1607_16.czi - ZEN 2.3 lite

The red rectangle indicates the part of the tumor, which is depicted above in Figure 11.

Notice the homogenous sarcomatous deeply basophilic tissue, surrounding the central frankly paler and more eosinophilic area of ischemic necrosis (red arrow), and deeply invading/ infiltrating the pre-existing fat tissue (white arrows).

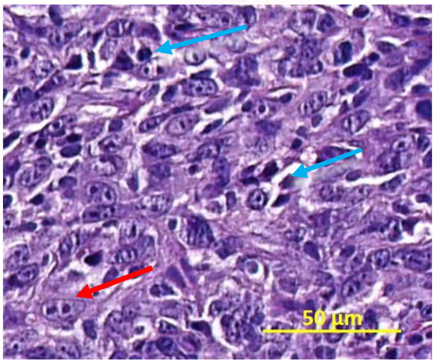

Source photo file: 2017-10-25 14_33_05-TPL811_17_HE_11975_TU_1607_16.czi - ZEN 2.3 lite

This detail of the neoplastic proliferation is located in the green rectangle of the topographical photo above

Neoplastic cells exhibit relatively low pleomorphism, occasionally prominent nucleoli (red arrow) rare mitoses and quite numerous infiltrating small lymphocytes (**blue arrows**). Mitoses are numerous (not shown).

JA-2042 cMDI: NOS sarcoma with hemangiosarcoma-like differentiation

JA-2042/0124-17: NOS sarcoma, with conserved hemangiosarcoma-like (angioid) regions

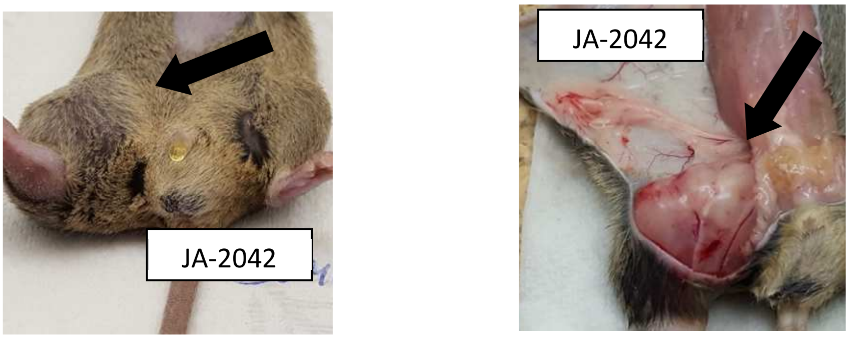

Figure 12: Low magnification (sub-gross aspect) of the tumor masses: **A** index tumor 2042-16 and **B**: established daughter tumor 124-17

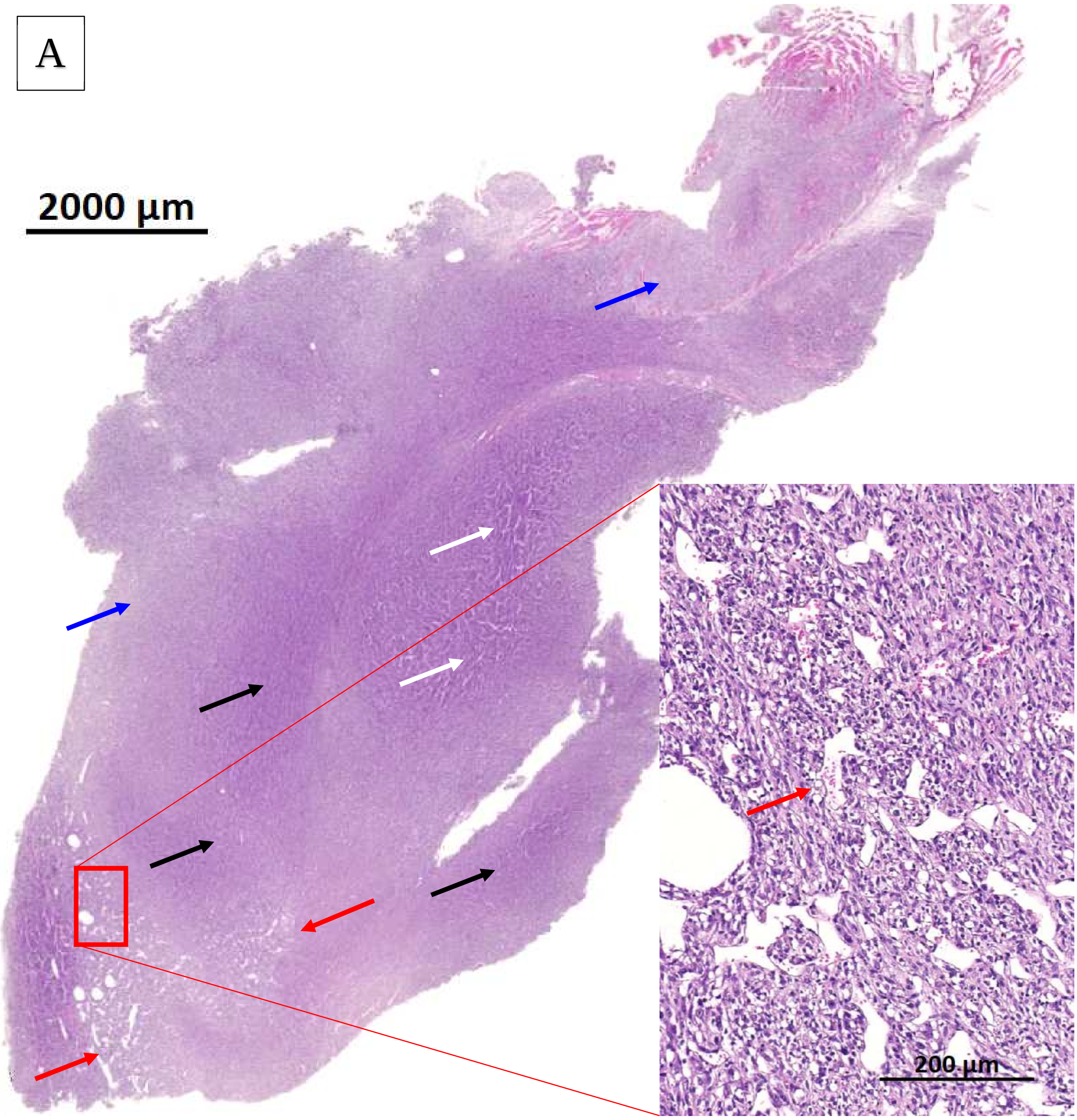

A: main mass of the index tumor showing 2 distinct cell density aspects, a higher more basophilic aspect (**black arrows**) and a lower paler and pinker aspect (blue arrows) corresponding to lower cell density with also possibly paler cytoplasm. In addition there are 2 small areas with dilated vessels containing red blood cells, mimicking an hemangioma or hemangiosarcoma differentiation (see red arrows and red rectangle in previous page)

Also, notice artefactual cracks in central areas of the sample (white arrows), suggesting lack of significant extracellular matrix.

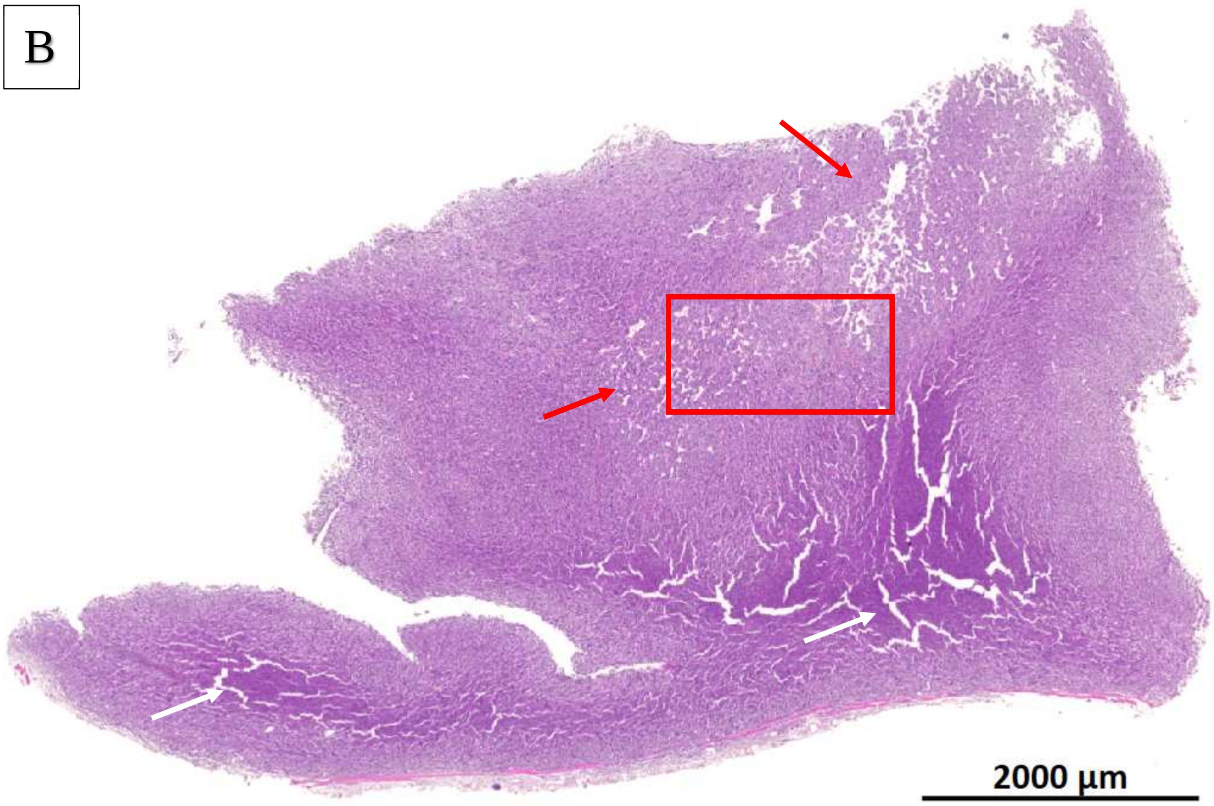

B: Sub-gross view of grafted JA-2042 into **124-17** mouse. The tissue similarity at this magnification is striking. Importantly, there are vascular profiles (see red arrow

There is also hemangiosarcoma differentiation, indicating remarkable phenotype stability (see red arrows and red rectangle). IHC is strongly advised here to document possible vascular differentiation.

Notice artefactual cracks in central areas of the sample (white arrows), suggesting lack of significant extracellular matrix.

Source file: C:\Users\TLEMARCHAND\Documents\ProQinase-Paper-Project\neue bilds\2019-01-24 15_15_40-#12551 124-17.czi - ZEN 2.3 lite.png: Extracted from TPL Study Phase Number 780/17

Figure 13: Comparison of different regions of index **2042-16** and derived **124-17**

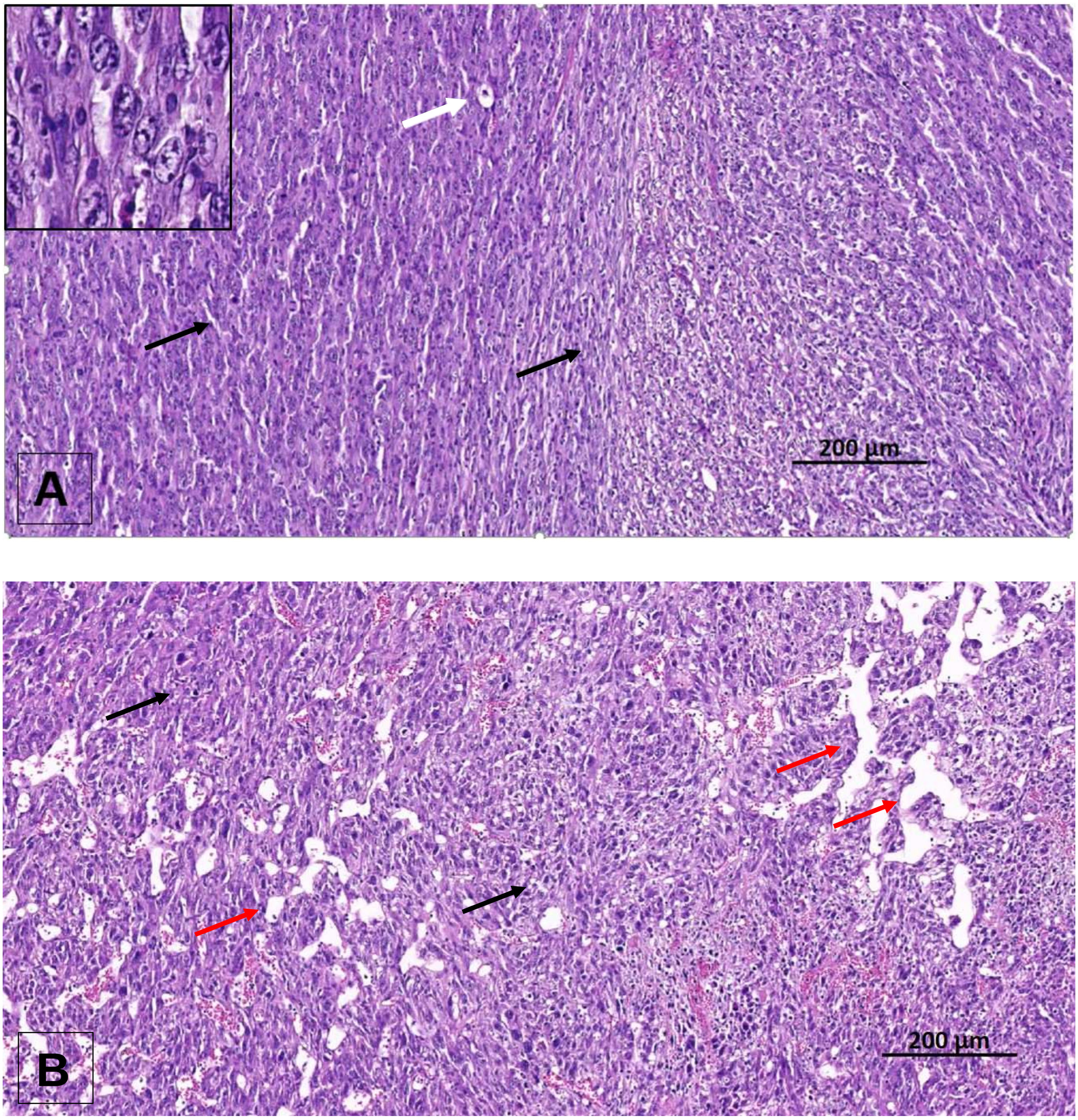

From index case: mouse ID number 2042-16. Strain CBA/J ♀. Induced with MCA.

Sarcoma, not otherwise specified (NOS), composed of interwoven bundles of spindle-shaped elongated cells with plump nuclei (see inset: neoplastic mesenchymal cells), with minimum anaplasia and clearer and denser areas (**black arrows**). Both the index and the established tumor either invades adjacent to blood vessels or exhibit vascular (angio-) differentiation, mimicking an hemangiosarcoma (**B**: red arrows). The index tumor also has rare spaces, related to single cell degeneration (**A**: white arrow). Notice the remarkable phenotypic stability, at least morphologically with H&E stain, when comparing the index **(A)** and derived **(B)** neoplasms. IHC is strongly advised here.

A: Image ID: 2017-10-24 17_39_33-11975_tu___2042-16___2042-16.czi - ZEN 2.3 lite – index case (mouse ID) **2042-16; B**: image ID: 2017-10-24 17_36_31-12551_124-17___Establish.czi - ZEN 2.3 lite – ID #**124-17**:

JA-2034 cMDI: NOS sarcoma within adipose tissue of the panniculus

JA-2034/0125-17: NOS sarcoma with adipose tissue of the panniculus

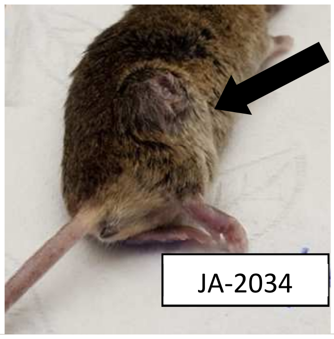

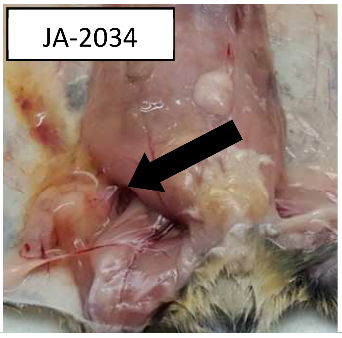

Figure 14: Chemically-induced neoplasms (study #11975): sarcoma, not otherwise specified, male CBA/J mouse 2034/16, MNU s.c.

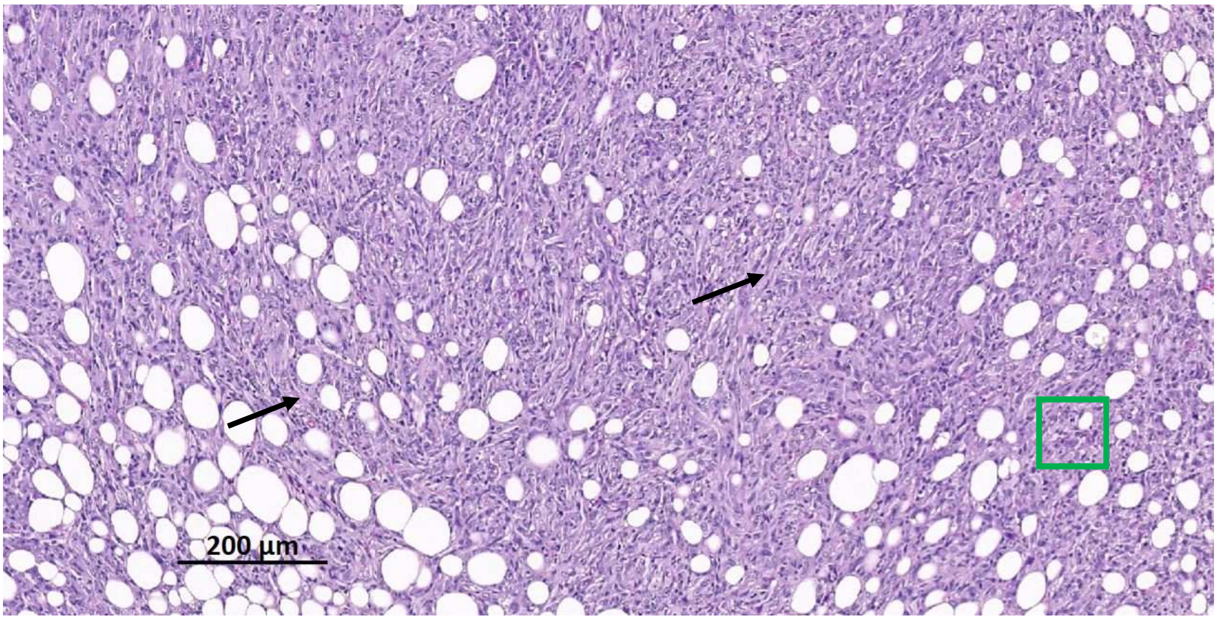

Source: 2017-10-25 13_51_00-TPL811_17_HE_11975_TU_2034_16.czi - ZEN 2.3 lite

Index case: mouse ID number 2034-14. Strain CBA/J sex: male (♂).

The adipose tissue of the panniculus is diffusely invaded by bundles of spindle-shaped cells, rather well differentiated, and of moderate anaplasia (see below; **black arrows**). Based on these samples, it is impossible to state whether the sarcoma NOS simply invades the panniculus or in fact exhibit liposarcomatous differentiation. Based on the normal aspect of the adipocytes, it is most likely that it is a sarcoma invading pre-existing normal adipose tissue?

Morphological diagnosis: Sarcoma NOS, MNU-induced, CBA/J mouse.

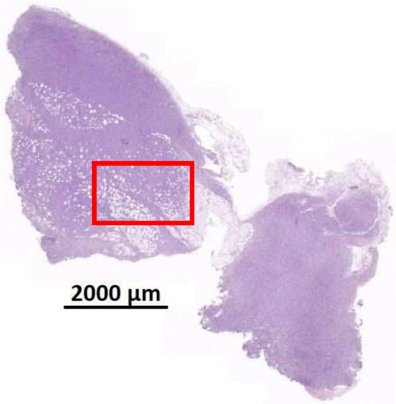

Source photo file: 2018-01-23 11_08_19-TPL811_17_HE_11975_TU_2034_16.czi - ZEN 2.3 lite

The red rectangle indicates the part of the tumor, which is depicted above (top large photo).

The tissue site of invasion is hypodermis (panniculus). Ischemic necrosis is absent

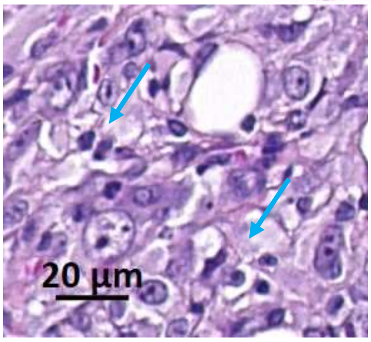

Source photo file: 2018-01-23 11_19_42-TPL811_17_HE_11975_TU_2034_16.czi - ZEN 2.3 lite

This detail of the neoplastic proliferation is located in the green rectangle of the top photo above

Neoplastic cells exhibit moderate pleomorphism (no bizarre anaplastic cells) and numerous small infiltrating lymphocytes (**blue arrows**).

Figure 15: Chemically-induced neoplasms (study #11975): sarcoma, not otherwise specified, male CBA/J mouse 0125/17, MNU s.c.

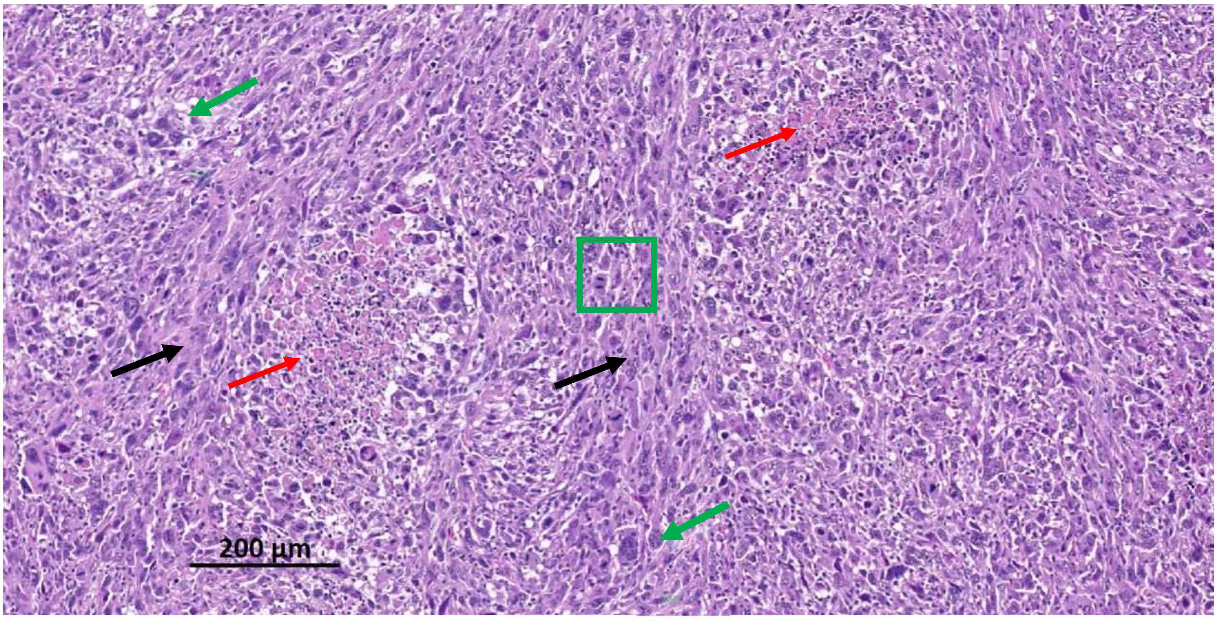

Source: 2017-10-25 13_52_20-TPL811_17_HE_11975_TU_0125_17.czi - ZEN 2.3 lite

Index case: mouse ID number 2034-14. Strain CBA/J sex: male (♂)

The invaded tissue type is unrecognizable. The sarcoma is composed of interlacing bundles of blastic poorly differentiated spindle-shaped cells (**black arrows**). When compared to the originator neoplasm (see Figure 14), there is an increased degree of anaplasia, more mitoses, with few bizarre giant cells (green arrows) and evidence of limited early ischemic necrosis, indicative of inadequate vascularization (red arrows).

Morphological diagnosis: Sarcoma NOS, MNU-induced, CBA/J mouse, 0125-17.

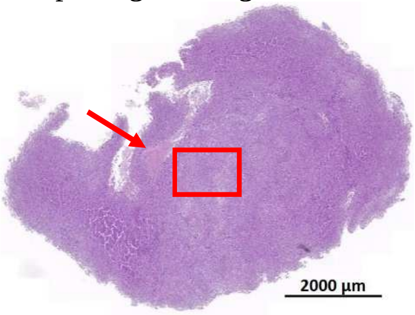

Source photo file: 2018-01-23 12_13_10-TPL811_17_HE_11975_TU_0125_17.czi - ZEN 2.3 lite

The red rectangle indicates the part of the tumor, which is depicted above.

Notice the homogenous sarcomatous tissue, with visible, slightly paler and more eosinophilic ischemic necrosis (red arrow).

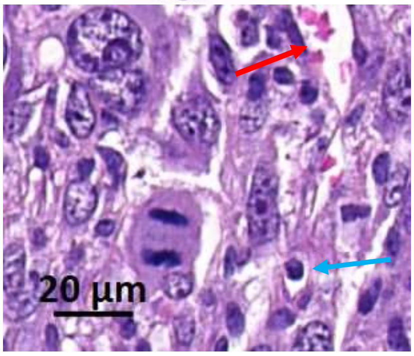

Source photo file: 2018-01-23 12_11_18-TPL811_17_HE_11975_TU_0125_17.czi - ZEN 2.3 lite

This detail of the neoplastic proliferation is located in the green rectangle of the top photo above

Neoplastic cells exhibit significant pleomorphism (with coarser nuclear chromatin and large nucleoli), and numerous mitoses and infiltrating lymphocytes (**blue arrow**). The blood vessel has very thin stroma (red arrow)

JA-2043 cMDI: NOS sarcoma with varying aspects from well differentiated to anaplastic

JA-2043/0074-17: NOS sarcoma with varying aspects from well differentiated to moderately

Figure 16: Macroscopic aspects of the tumor JA-2043

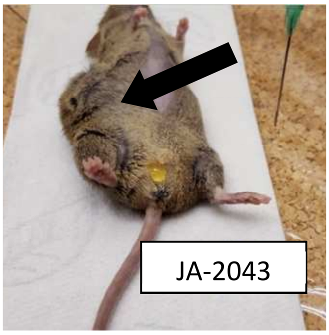

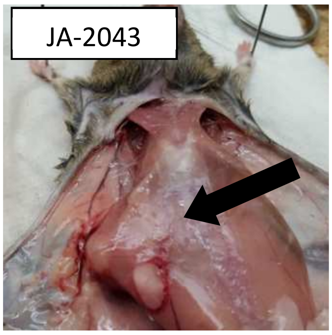

Figure 17: High magnification aspects of the index tumor 2043-16: anaplastic

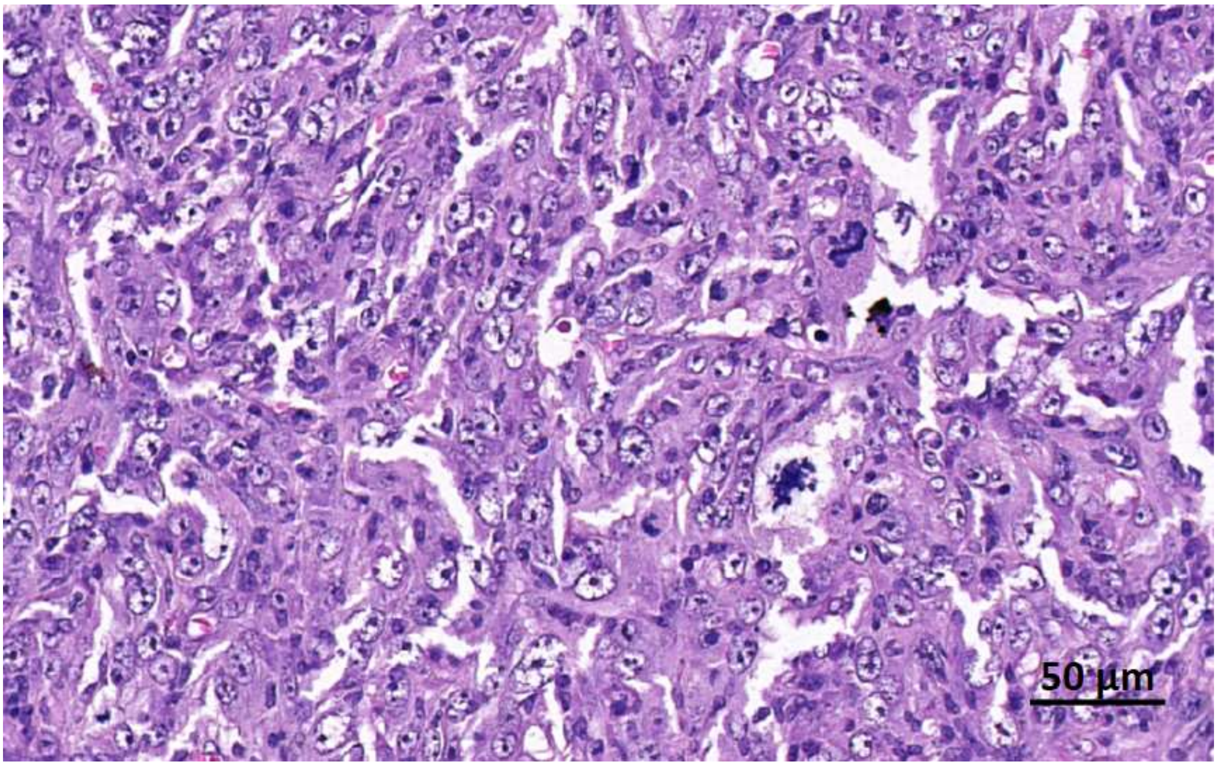

The sarcomatous tissue is characterized by high variability of size and shape of cells and nuclei ("anisopoikilocytosis" and "anisopoikilokaryosis"), vesicular nuclei and multinucleated cells with condensed nuclei and/or abnormal /abortive mitoses or "mitonecrosis" (**see black arrows**) – the section has some artefactual cracks. This is an example of moderate to low differentiation, with a rather anaplastic aspect. This aspect is on the top specimen of Figure 18.

Tumor diagnosis (HE evaluation) - with description	Other comments
Sarcoma NOS, varying aspects, from well differentiated to moderately anaplastic; no giant cells	- deeply invading the skin up to the upper dermis<br>- many anaplastic features (abnormal mitoses, large vesicular nuclei) but no giant multinucleate cells

Figure 18: Low magnification (sub-gross aspect) of the tumor masses of the index tumor **2043-16**

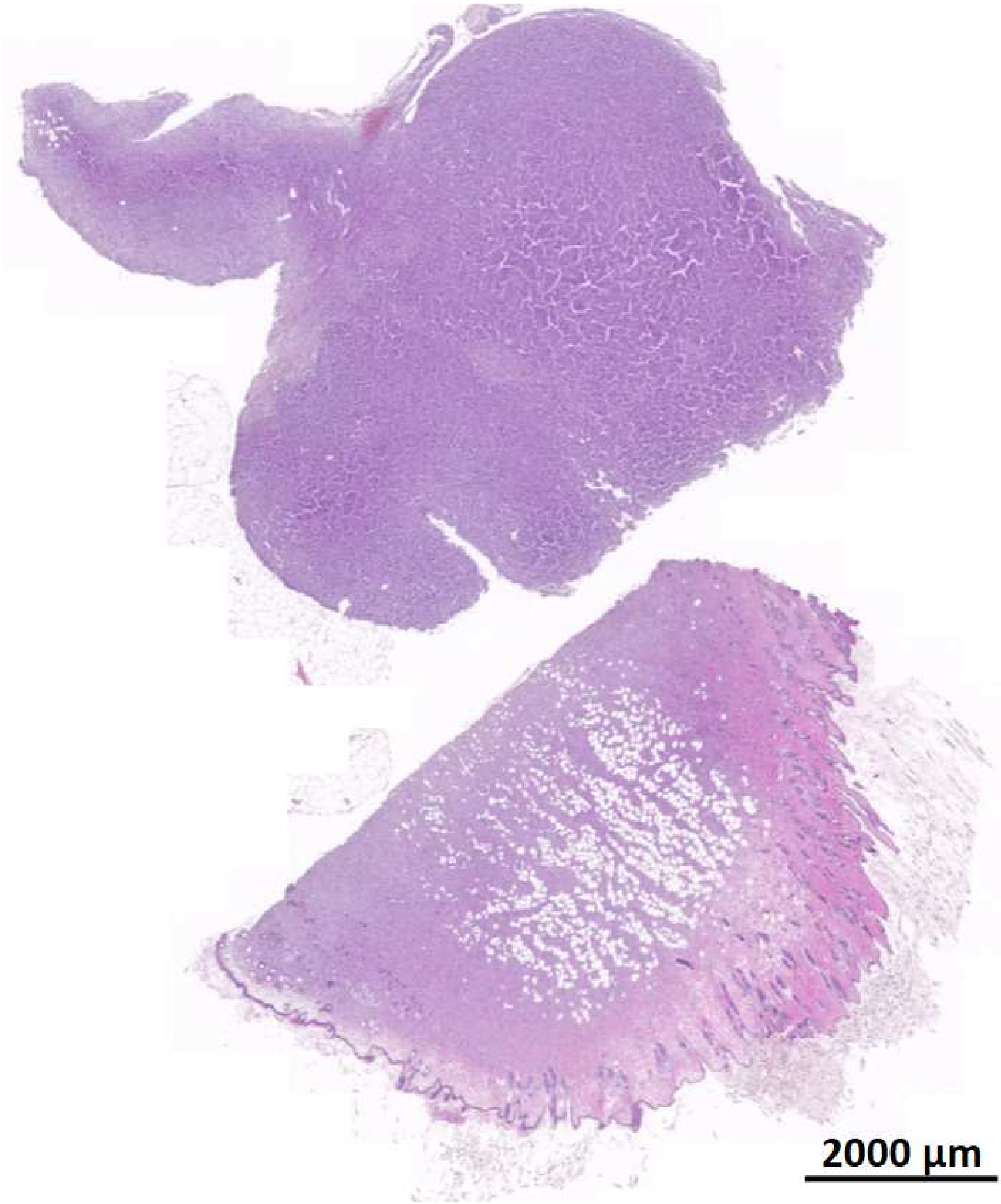

Figure 19: High magnification aspects of the index tumor 2043-16: fusiform aspects

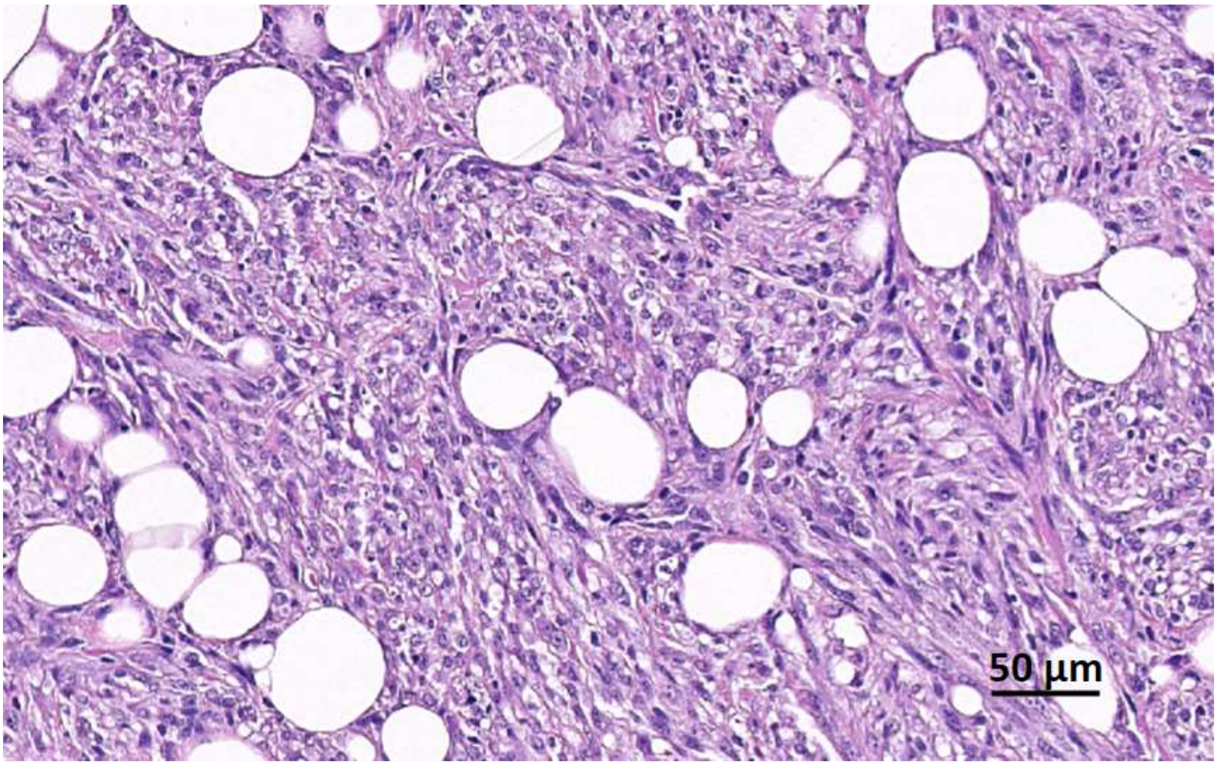

The tumor tissue is characterized by interwoven basket weave bundles of rather well differentiated fibroblasts, which however finely invade and dissect the pre-existing sheets of normal adipocytes of the subcutis.

This aspect is on the low specimen of Figure 18. Extracted from TPL Study Phase Number 811/17

Figure 20: Sub-gross and higher views of grafted JA-2043 into 0074-17 mouse. Tissue similarity with top index tumor at these magnifications is striking. N is central ischemic necrosis of the tumor

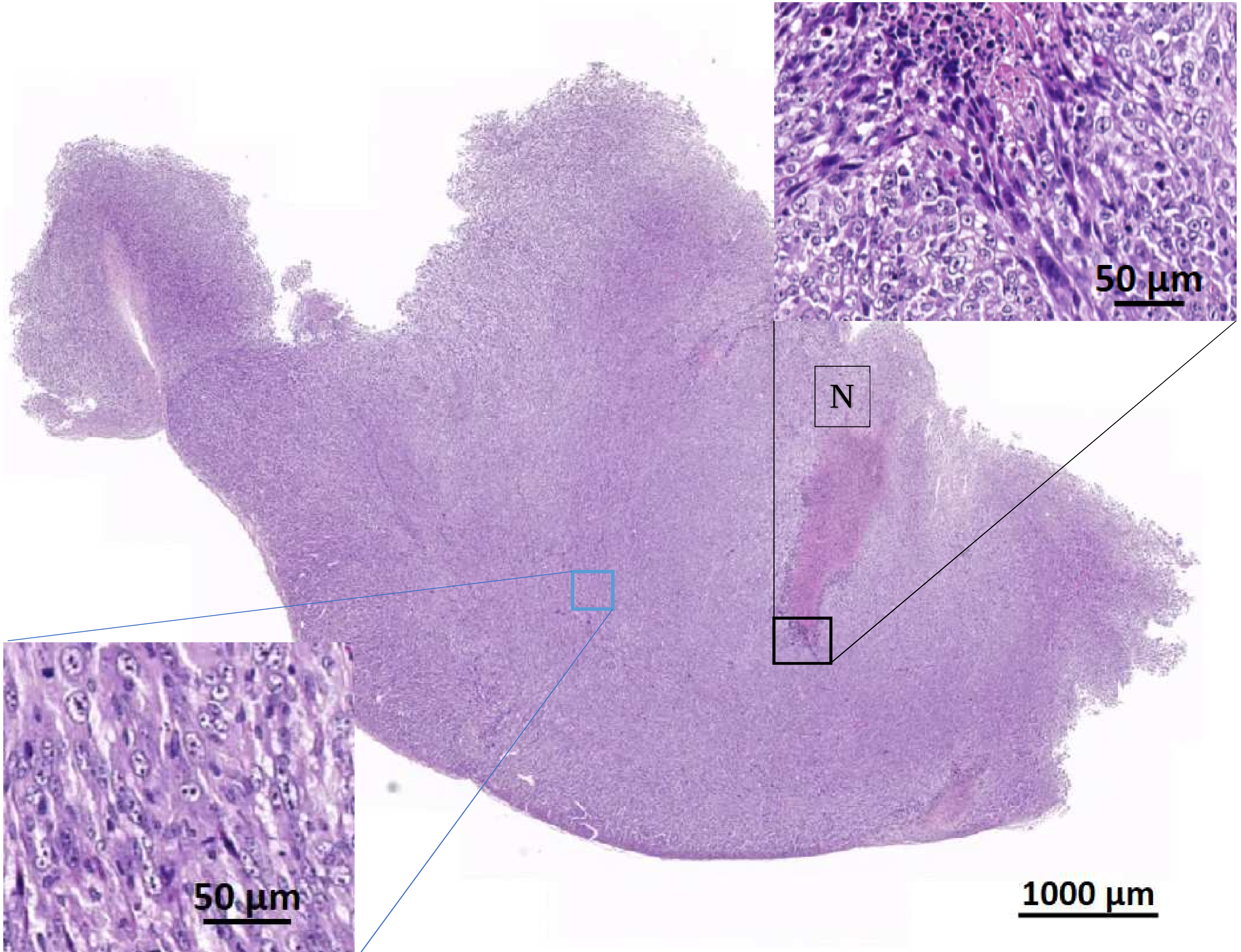

JA-2044 cMDI: Spinocellular carcinoma (epidermoid carcinoma)

JA-2044/1418-16: Spinocellular carcinoma (epidermoid carcinoma) which is an epithelial (epidermal and/or adnexae) neoplastic proliferation

no *in situ* Foto

Figure 21: Chemically-induced neoplasms (study #11975): spinocellular carcinoma, male CBA/J mouse 2044/16, MCA s.c.

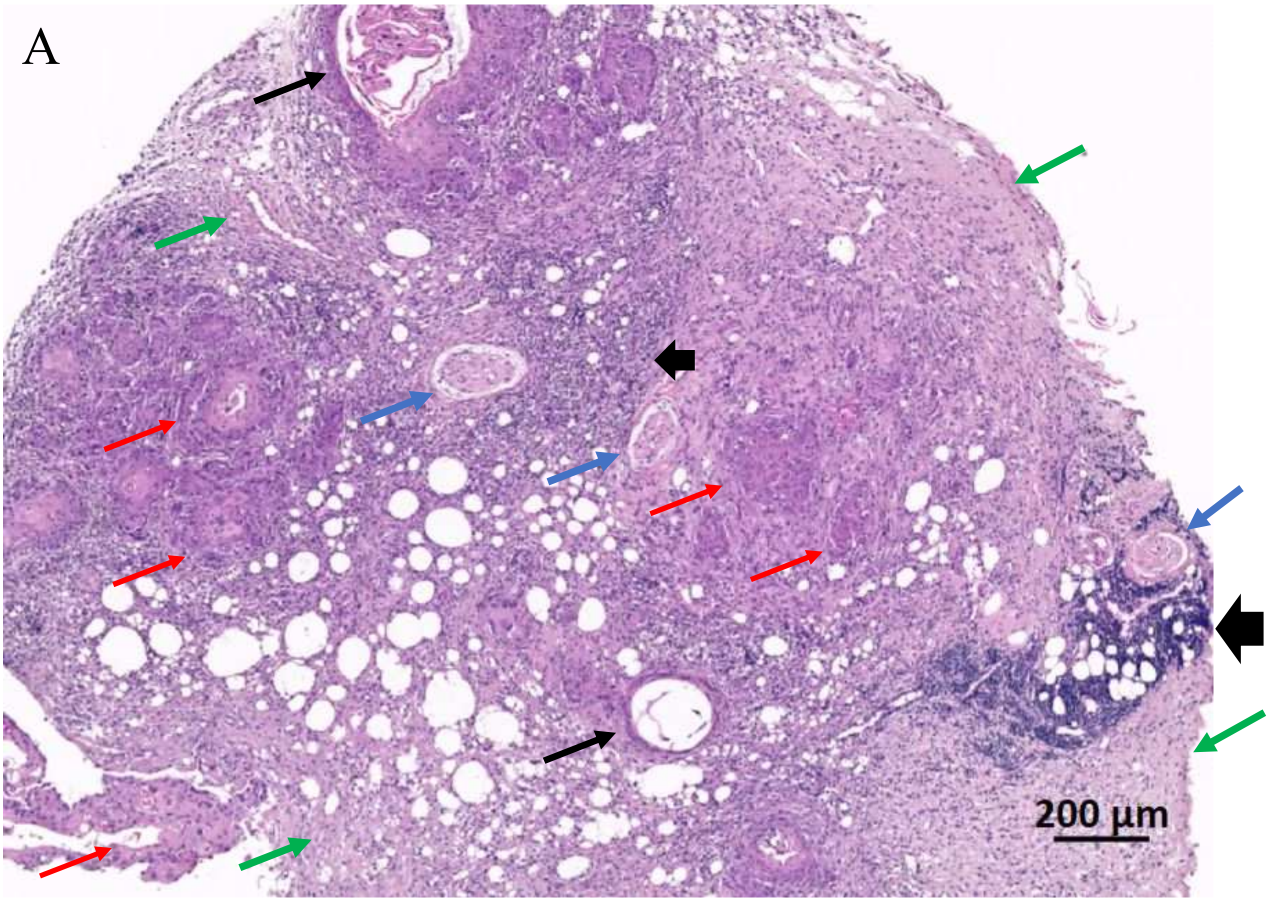

<u>Source</u>: 2017-10-25 13_37_55-TPL811_17_HE_11975_TU_2044_16_11_10_16.czi - ZEN 2.3 lite

<u>Index case</u>: mouse ID number 2044-14. Strain CBA/J sex: male (♂)

A: This is an epithelial neoplastic proliferation from the skin, characterized by epidermal differentiation and keratin production, which induces intense inflammation, with a pronounced lymphoid component (**arrowheads** ⬅), as well as fibrosis (**green arrows**).

It is characterized by cords and nodular elements of keratinocytes (**red arrows**) with central laminar keratinization, often forming central cysts with occasional horn pearls (**black arrow**). The neoplasm is entrapping a nerve (blue arrows).

Morphological diagnosis: Spinocellular carcinoma, skin, MCA-induced, CBA/J mouse.

B:

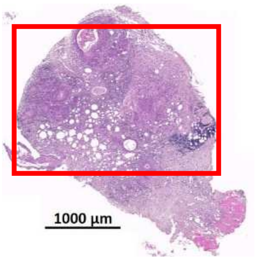

B: Thumbnail source photo file: 2018-01-23 10_02_25-TPL811_17_HE_11975_TU_2044_16_11_10_16.czi - ZEN 2.3 lite

The red rectangle indicates the part of the neoplasm, which is depicted above

Cutaneous muscle

Figure 22: Chemically-induced neoplasms (study #11975): spinocellular carcinoma, male CBA/J mouse 1418/16, MCA s.c.

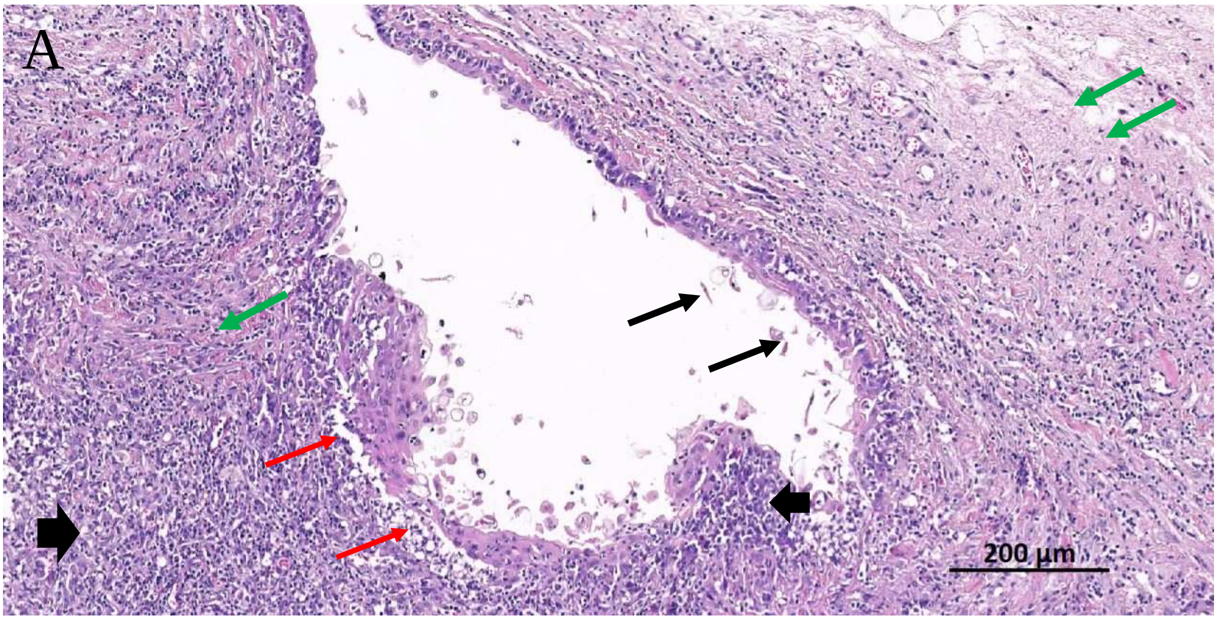

Source: 2017-10-25 13_43_16-TPL811_17_HE_11975_TU_1418_16.czi - ZEN 2.3 lite

Index case: mouse ID number 2044-14. Strain CBA/J sex: male (♂)

A: The epithelial proliferative component of this tumor is again the neoplastic epidermal spinocellular (keratocytic) elements (**red arrows**) mostly forming cystic spaces, in which the dyskeratotic keratinization occurs without horn pearl formations (**black arrows**). However, the majority of the tumor (see below panel B) is composed of florid (desmoplastic) fibrosis (**green arrows**) and intense lymphocytic infiltrative ( ) stromal elements, underlining the similarity with the neoplasm depicted in Figure 21.

Morphological diagnosis: Spinocellular carcinoma, skin, MCA-induced, CBA/J mouse

B: Low magnification of tumor aspect overview (thumbnail):

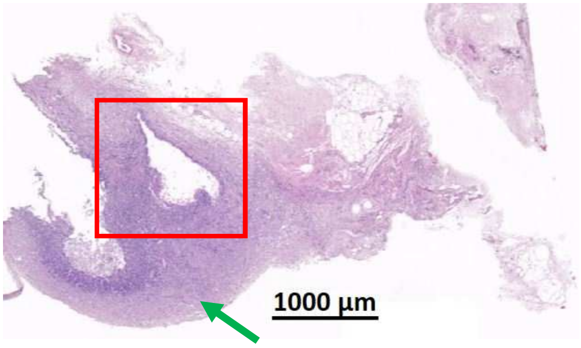

The red rectangle indicates the part of the neoplasm, which is depicted above

The vast majority of the tumor on the section examined is composed of desmoplastic and highly inflamed stroma (green arrow).

Source photo file: 2018-01-23 10_17_10-TPL811_17_HE_11975_TU_1418_16.czi - ZEN 2.3 lite

Note: ID = identification code

Animal	Spec. ID (loc)	originator	Tumor diagnosis (HE evaluation)	Comments
2044-16	#11975_1 2044/16 11.10.16	2044-14	**spinocellular carcinoma**	intense lymphoid and neutrophilic inflammation
1418-16	#11975_Tu 1418/16	2044-14	**spinocellular carcinoma**	high levels of inflammation; glandular differentiation

JA-2017 cMDI: NOS sarcoma varying from well differentiated to moderately anaplastic; no giant cells

JA-2017/1573-16: NOS sarcoma varying from well differentiated to moderately anaplastic; no giant cells.
The re-transplanted tumors grow only in SCID/bg

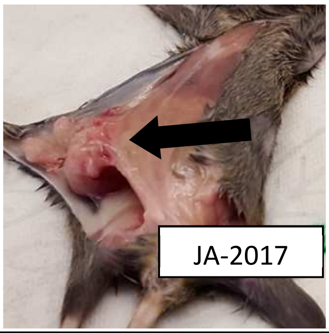

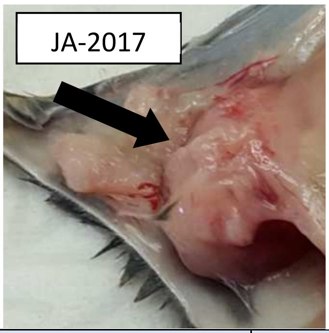

Animal	Spec. ID (loc)	originator	Tumor diagnosis (HE evaluation) - with comments	Comments for next step(s)
2017-16	#11975_Tu 2017/16	2017-14	Sarcoma NOS, varying aspects, from well differentiated to moderately anaplastic; no giant cells	Na – see Figure 24
1573/16	#11975_Tu 1573/16	2017-14	Sarcoma NOS, varying aspects, from well differentiated to moderately anaplastic; no giant cells	abundant ischemic necrosis – see Figure 25 and Figure 26

Figure 23: Originator tumor of the JA-2017: **2017-16** (see next page)

There is presence of bundles of relatively homogenous blastic spindle-shaped cells (lower left inset), which destroy and dissect the pre-existing skeletal striated muscles (upper right inset)
Please see next page (landscape)
(see next page)

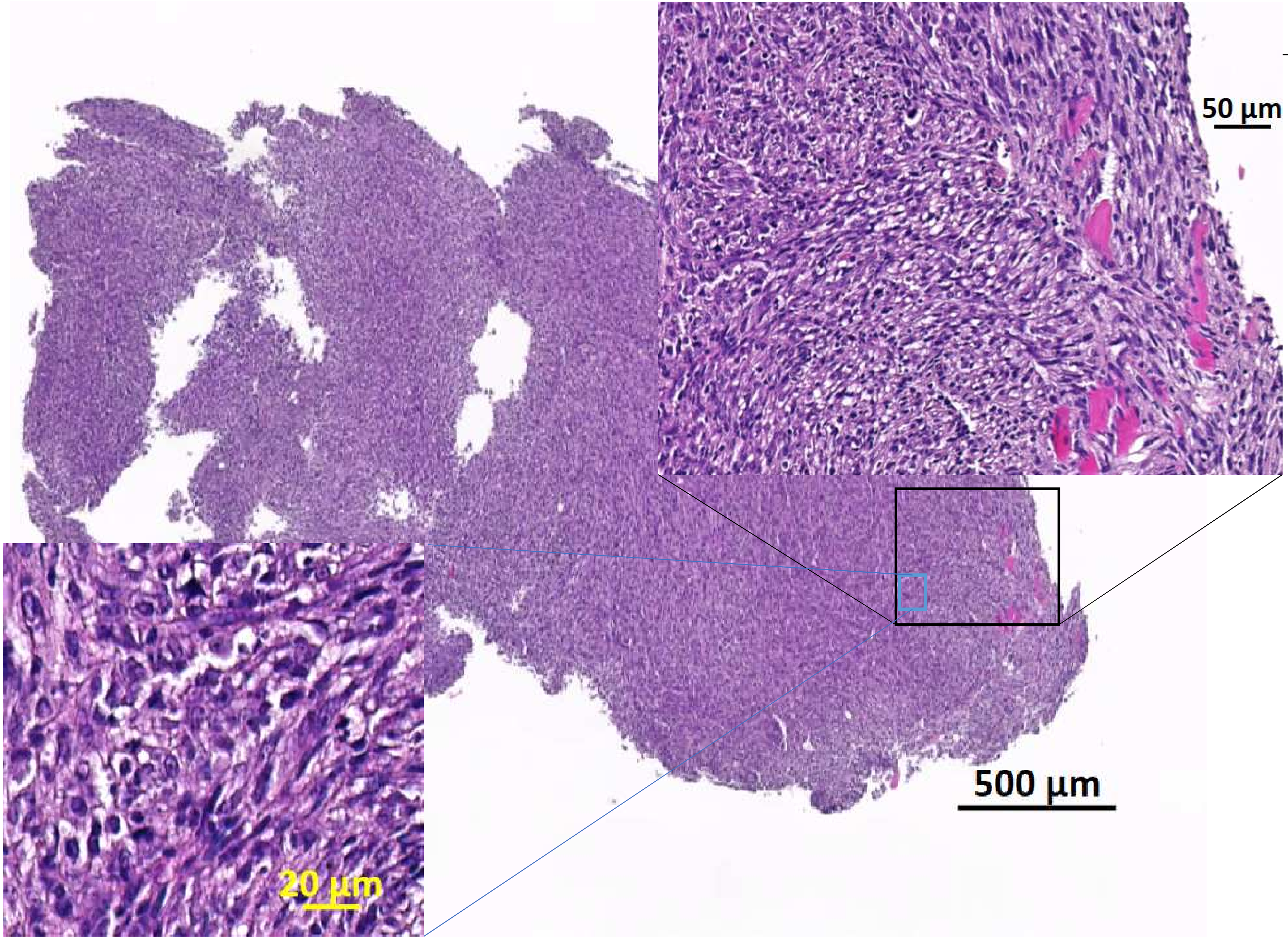
50 µm
500 µm
20 µm

cancers MDPI

Figure 24: Low magnification aspect of the derived **tumor 1573-16** grafted with **JA-2017**

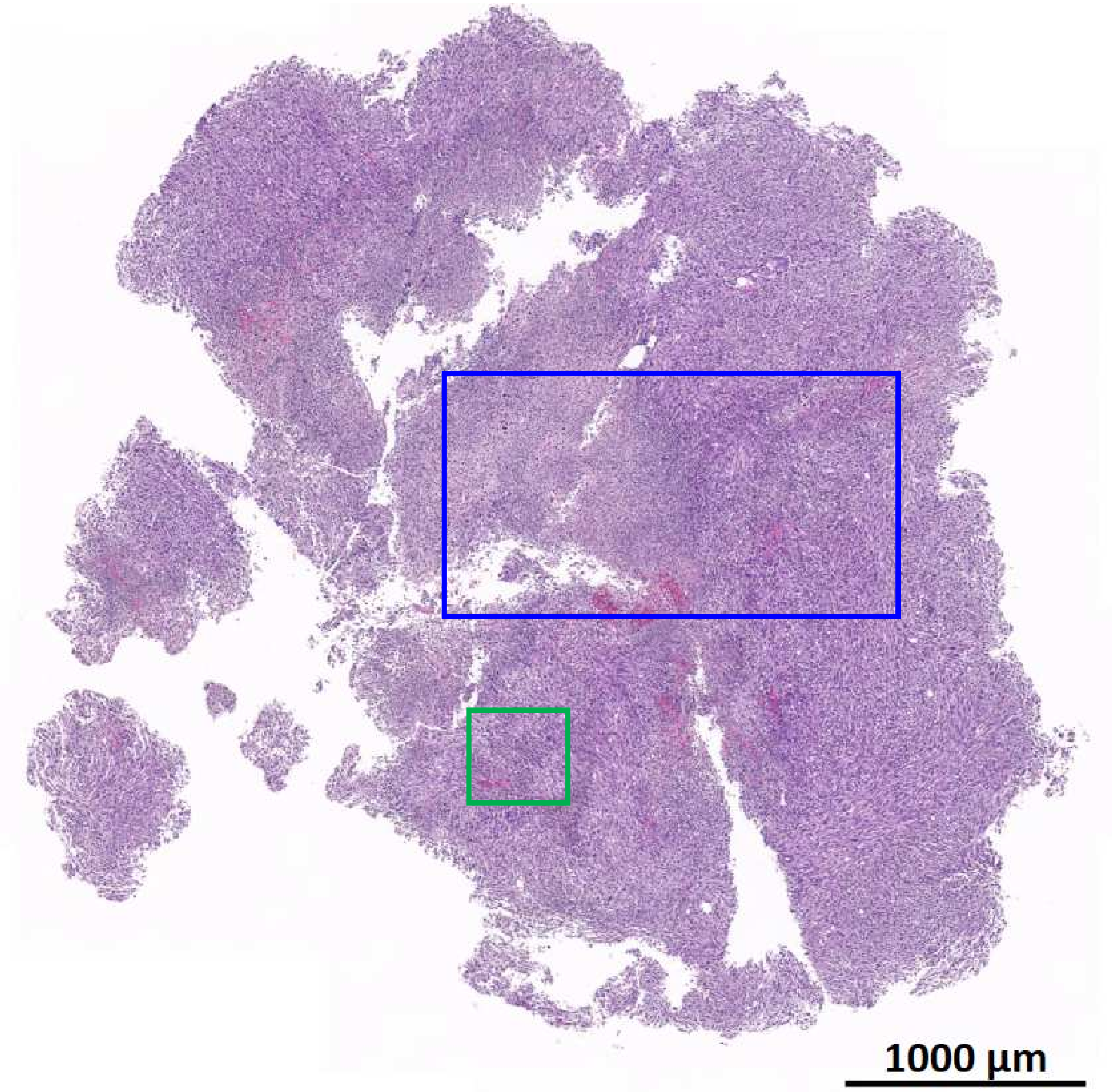

The tumor is fragmented and has several areas of necrosis of tumor tissue separated by a relatively dense line of intact and degenerating inflammatory cells, encompassing neutrophils and mononuclear cells, including relatively numerous small lymphocytes

Figure 25: Tumor **1573-16**: High magnification view of the region in the Green square

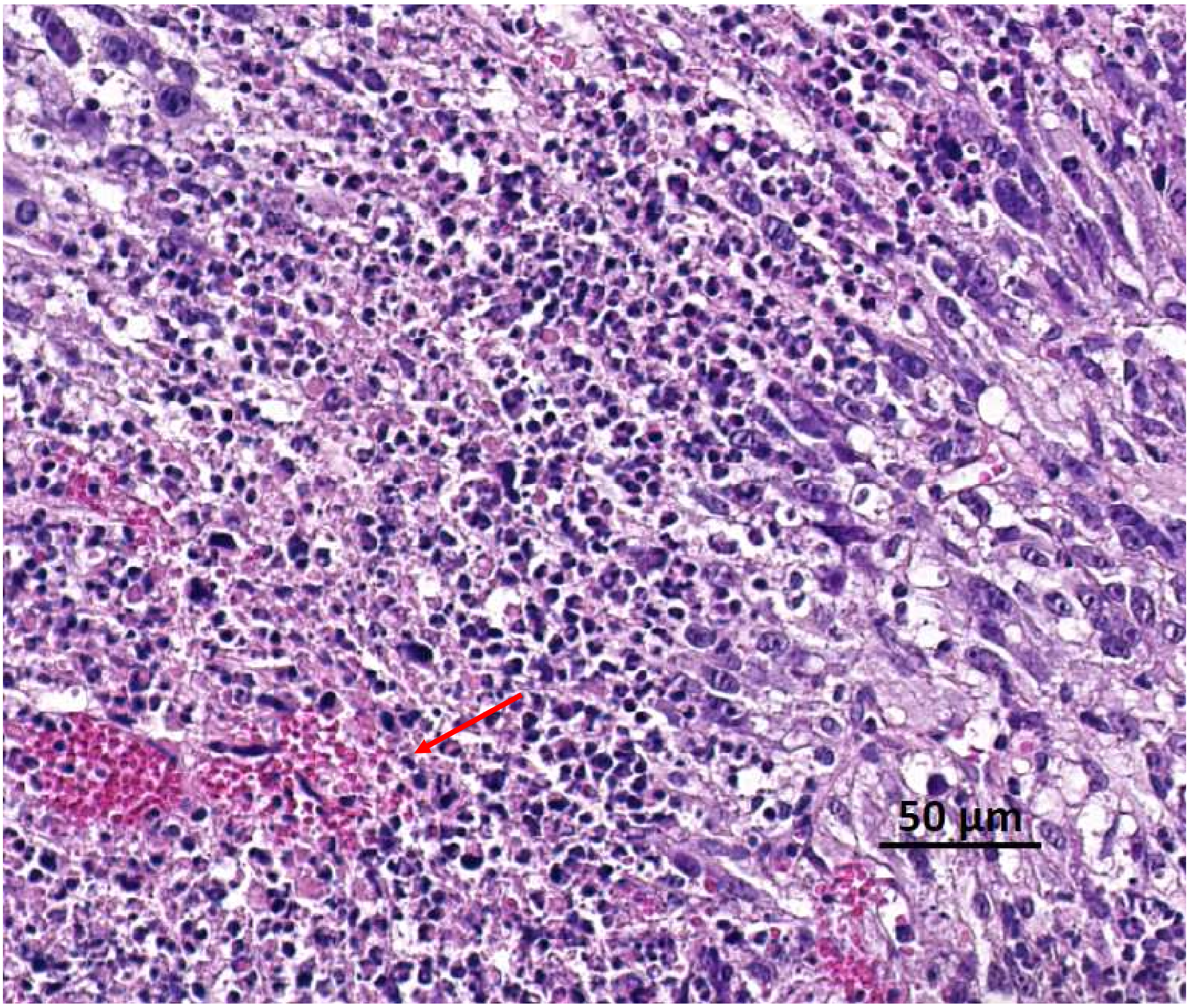

The inflammatory cells composing the interface of intact and degenerated neoplastic tissue (with hemorrhage) is mainly composed of intact and degenerated granulocytes, with focal presence of mononuclear cells (see inset below next page). In central ischemic necrosis there is associated red blood cell extravasation (red arrows).

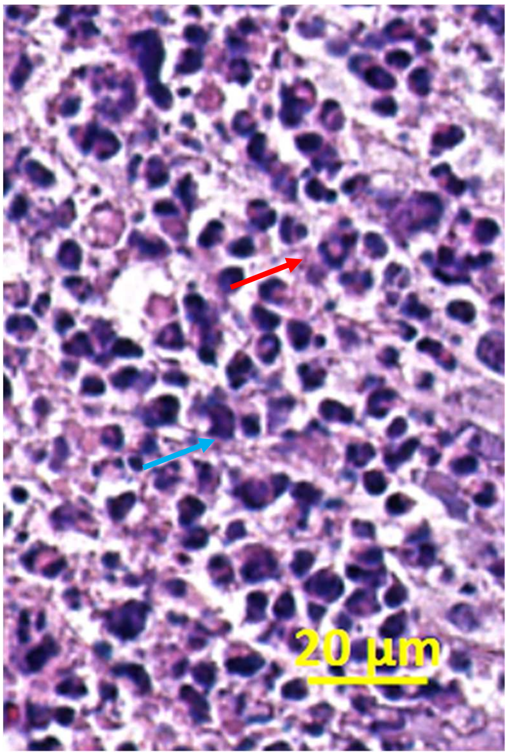

Detail of the Figure 25 depicting the cytological details of the inflammatory infiltration at the interface of the intact and degenerated neoplastic tissue, with mainly intact and degenerated granulocytes (red arrow). Mononuclear cells suggesting monocytes and/or lymphocytes are relatively rare (blue arrow)

Figure 26 : Tumor 1573-16: Medium magnification view of the region in the large blue rectangle from figure 24

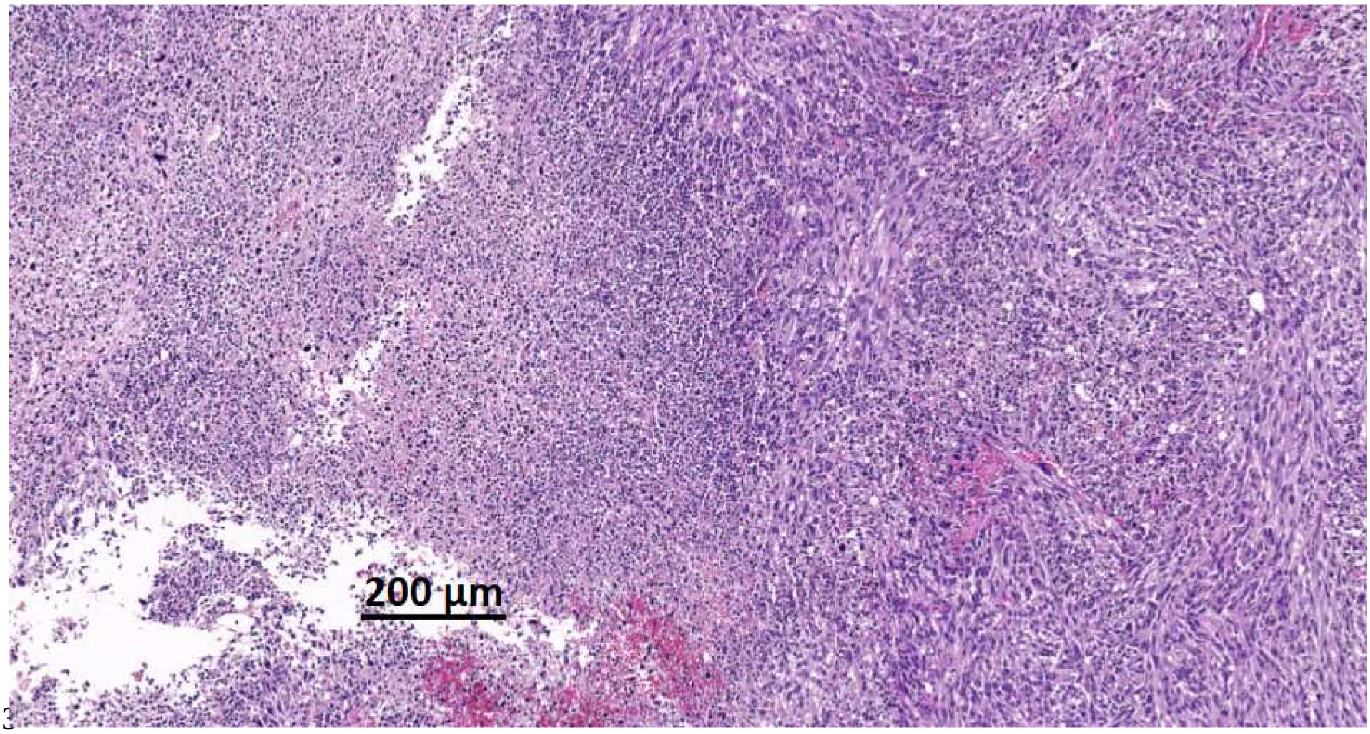

Areas of necrosis are numerous and coalescing with hemorrhage and abundant leukocytes: Extracted from TPL Study Phase Number 811/17

Abbreviation list

AdK = ADC	Adenocarcinoma
DDX. = Diff. Dx.	Differential diagnosis
BALT	Bronchus associated lymphoid tissue
Bein	Leg
Brustdrüse = rü	Mammary gland
Darm	Intestinal
H&E = HE	Hematoxylin and eosin
ID	Identification
IHC	Immunohistochemistry
K	Carcinoma
Leb	Liver (Leber)
MCA	Methylcholanthrene
MNU	1-methyl-1-nitrosourea
MGCs	Multinucleate giant cells (syncytial macrophages or neoplastic cells)
mz	Spleen (Milz)
NA	Not applicable
Niere = ni = nie	Kidney
NOS	Not otherwise specified
PND	Post-natal Day
S	Sarcoma
s.c.	Sub-cutaneous
SCID	Severe Combined Immuno-Deficiency
Spec.	Specimen
TIL	Tumor infiltrating lymphocytes

© 2018 by the authors. Submitted for possible open access publication under the terms and conditions of the Creative Commons Attribution (CC BY) license (http://creativecommons.org/licenses/by/4.0/).
